# Uncontrolled Thyroid during Pregnancy Alters the Circulative and Exerted Metabolome

**DOI:** 10.3390/ijms23084248

**Published:** 2022-04-12

**Authors:** Charalambos Fotakis, Giorgos Moros, Anna Kontogeorgou, Nicoletta Iacovidou, Theodora Boutsikou, Panagiotis Zoumpoulakis

**Affiliations:** 1 Institute of Chemical Biology, National Hellenic Research Foundation, 48 Vas. Constantinou Ave., 11635 Athens, Greece; bfotakis@yahoo.com (C.F.); geomoro@hotmail.gr (G.M.); 2 Neonatal Department, Aretaieio Hospital, Medical School, National and Kapodistrian University of Athens, 11528 Athens, Greece; annakon.doc88@gmail.com (A.K.); niciac58@gmail.com (N.I.); 3 Department of Food Science and Technology, University of West Attica, Ag. Spyridonos, Egaleo, 12243 Athens, Greece

**Keywords:** thyroid disorder, thyroid hormones, metabolic pathways, early diagnosis, fetal growth and development, NMR metabolomics, chemometrics

## Abstract

Normal levels of thyroid hormones (THs) are essential for a normal pregnancy outcome, fetal growth and the normal function of the central nervous system. Hypothyroidism, a common endocrine disorder during pregnancy, is a significant metabolic factor leading to cognitive impairments. It is essential to investigate whether patients with thyroid dysfunction may present an altered circulative and excreted metabolic profile, even after receiving treatment with thyroxine supplements. NMR metabolomics was employed to analyze 90 serum and corresponding colostrum samples. Parallel analyses of the two biological specimens provided a snapshot of the maternal metabolism through the excretive and circulating characteristics of mothers. The metabolomics data were analyzed by performing multivariate statistical, biomarker and pathway analyses. Our results highlight the impact of hypothyroidism on metabolites’ composition during pregnancy and lactation. Thyroid disorder causing metabolite fluctuations may lead to impaired lipid and glucose metabolic pathways as well as aberrant prenatal neurodevelopment, thus posing a background for the occurrence of metabolic syndrome or neurogenerative diseases later in life. This risk applies to not only untreated but also hypothyroid women under replacement therapy since our findings in both biofluids framed a different metabolic phenotype for the latter group, thus emphasizing the need to monitor women adequately after treatment initiation.

## 1. Introduction

Thyroid hormones (THs) are essential for growth and development in children and adolescents and have a fundamental role in fetal and neonatal brain development and body growth [1,2].

At the cellular level, THs participate in the positive regulation of the carbohydrate metabolism, lipid catabolism and the stimulation of protein synthesis in a wide variety of cells [2]. THs are mainly involved in the regulation of the metabolic rate; thus, abnormal thyroid functioning results in decreased metabolic activity, i.e., hypothyroidism is implicated in impaired mental and physical activity [2,3]. In particular, when a thyroid hormone deficiency occurs, the brain in the fetal and postnatal development stages could be compromised, resulting in a retarded maturation, intellectual deficits and neurological impairments [4]. In fact, a number of pioneering studies by Man et al. [5] and Haddow et al. [6] and newer studies by Rovet et al. [7] and Pop et al. [8] showed that children born to mothers with hypothyroidism carried a significantly increased risk of impairments of IQ scores, neuropsychological developmental indices and learning abilities. Children born to untreated hypothyroid women had IQ scores that were seven points below the mean IQs of children born to healthy women and women treated with thyroxine. This risk applies not only to children of untreated women but also to those of women with suboptimal treatments. A study by Rovet et al. [7] found that such children had mild defects in global intelligence, while their visual–spatial abilities, language, fine motor performance and preschool abilities were unaffected. Therefore, hypothyroidism constitutes not only a common endocrine disorder during pregnancy but also a significant metabolic factor for cognitive impairment. Multiple maternal and fetal adverse outcomes, such as miscarriage, pre-eclampsia, eclampsia, placental abruption and preterm delivery, are linked to impaired maternal thyroid dysfunction [9,10,11].

A pathophysiological condition results in specific and characteristic changes in the biochemical compositions of biological fluids. Metabolomics constitutes an ideal bioanalytical holistic approach to monitoring such changes [12]. Nuclear magnetic resonance (NMR) spectroscopy is a powerful analytical platform that has been widely employed in metabolomics studies to highlight metabolic signatures due to prenatal disorders, pregnancy complications and nutrition and to even detect early disease factors [13,14,15,16,17]. Todate, NMR metabolomics is mainly involved in thyroid cancer research [18,19,20,21], while literature utilizing the NMR metabolomics approach in research on thyroid dysfunction is scarce. A few recent studies utilized the metabolic patterns of serum samples to either provide insights into the metabolic intermediates between GHT (gestational hypothyroidism) and its related disease risks [22] or to indicate systematic differences between hyperthyroidism, hypothyroidism and control groups in patients with autoimmune thyroid dysfunction [23]. Another study utilized ^1^H NMR-based metabolomics on patients with newly diagnosed hypothyroidism and postulated that metabolic changes may persist even after the normalization of the serum levels of FT3, FT4 and thyroid-stimulating hormone (TSH) [24]. While a lot of attention has been focused on the adverse fetal outcomes consequent to hypothyroidism, attention is also being gradually directed toward the adverse maternal outcomes of this disorder. Thyroid dysfunction causing metabolite fluctuations may lead to impaired lipid and glucose metabolic pathways as well as aberrant prenatal neurodevelopment, thus posing a background for the occurrence of metabolic syndrome or neurogenerative diseases later in life [25,26,27,28].

Based on the above, we hypothesized that patients with thyroid dysfunction may present an altered metabolic profile, even after receiving treatment with thyroxine.

The rational compartment for the aforementioned NMR metabolomics studies was serum, a biofluid best reflecting the body’s responses to nutrition, disease, treatment and environmental factors. Another biofluid gaining increasing attention in studies on the effects of hypothyroidism on lactation is colostrum, which is produced until the 7th day postpartum and constitutes the optimal food for all newborns. It contains bioactive compounds that establish the immune protection of infants, thus providing multiple advantages for infants’ mental and physical development [29,30]. A number of studies have documented that hypothyroidism has an impact on both the quantity of human milk and the milk’s protein composition [31,32]. Evidently, another question arises regarding the impact of maternal thyroid disease on the composition of milk and possible implications for infants.

On these grounds, with the current study, we aimed at exploring whether distinctive serum metabolic patterns are related to thyroid disorder and at identifying whether the effects of thyroid disease during pregnancy affect the composition of colostrum. Maternal serum and colostrum samples were collected prospectively, aspiring to bridge the metabolic aberrations and possible comorbidities. Parallel analyses of the two biological specimens provided a snapshot of the maternal metabolism through the excretive and circulating characteristics of mothers. The application of NMR metabolomics facilitated the identification of metabolites in these biological specimens and provided relevant biomarkers to improve diagnostic accuracy, define prognoses and monitor treatment efficacy. Our results highlight the impact of hypothyroidism on metabolites’ composition during pregnancy and lactation, further prompting hypothyroidism’s early diagnosis and management. 

## 2. Results and Discussion

### 2.1. Circulatory Metabolome

Serum provides a rational compartment to interpret metabolite variations; therefore, our first step in our study was the metabolomics analysis of maternal serum blood samples. 

A characteristic NMR spectrum acquired with a CPMG (Carr-Purcell-Meiboom-Gill) pulse sequence of the serum blood is displayed in Figure S1 with annotations on the identified metabolites.

The maternal thyroid function of the participants was recorded at one time point for each pregnancy in the first trimester. The gestational hypothyroidism cases had either a first-trimester TSH > 2.5 mIU/mL with or without the presence of anti-thyroid autoantibodies (antiTPOs) or positive antiTPOs in the context of pre-existing thyroid disease. The mothers with disordered thyroid statuses either were receiving preconceptional thyroxine due to hypothyroidism or developed hypothyroidism within the first trimesters of their pregnancies, thus initiating thyroxine treatments (Table S1). There is evidence that 24–49% of women treated with levothyroxine (LT4) prior to conception still have elevated TSH levels at their first prenatal visit [33]. This could possibly be attributed to insufficient dosage adjustments to meet gestational requirements or to a lack of thyroid-function follow-ups at frequent intervals.

A PCA model with two components after the exclusion of outliers was computed for the serum samples, providing an overview and revealing trends of the samples’ clustering related to the presence of thyroid disorder (Figure S2). A clustering along the second principal component probed to a differentiation between the two groups. Interestingly, the control serum samples tended to cluster toward the third and fourth quadrants, while the samples of the mothers with thyroid dysfunction were mainly located in the first and second quadrants. 

**Figure 1:**
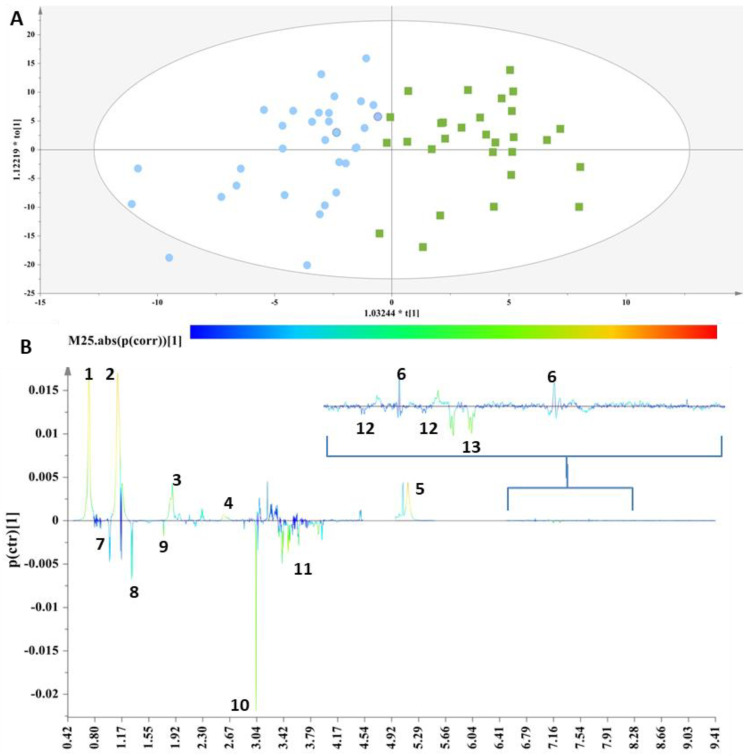
(**A**) OPLS-DA model; A = 1 + 1, N = 62; R^2^X(cum)= 0.61, R^2^Y(cum) = 0.62 and Q^2^(cum) = 0.45. Green squares = samples with thyroid disorder and blue circles= control samples. (**B**) S-line plot (1: cholesterol-VLDL, 2:LDL2/VLDL2, 3: n-acetylated glycoproteins, 4: methionine (2-amino-4-(methylsulfanyl)butanoic acid), 5: unsaturated lipid, 6: 1-methylhistidine, 7: valine (2-amino-3-methylbutanoic acid), 8: alanine (2-aminopropanoic acid), 9: acetic acid, 10: methanol, 11: glycerol, 12: tyrosine(2-amino-3-(4-hydroxyphenyl)propanoic acid) and 13: L-phenylalanine (2-amino-3-phenylpropanoic acid).

These two groups were then subjected to supervised analyses to resolve the metabolic variations, further analyze the data and elucidate potential markers. Class information with respect to the thyroid status of each sample was embedded into OPLS-DA models to pinpoint the metabolites responsible for the discrimination. For this purpose, a workset utilizing the samples included in the PCA model (Figure S2) was used as a training set to produce the first OPLS-DA model (Figure 1A),and then a prediction set of 10 samples and a validation set of 18 samples complemented the final OPLS-DA model (Figure S3A). The extracted OPLS-DA models correctly classified 75.76% of the serum samples.

The discrimination between the two clusters was evident along the first component (Figure 1A), and the key metabolites, which exhibited a strong correlation with the thyroid samples as depicted in the S-line plot (Figure 1B), were unsaturated lipids, cholesterol-VLDL, LDL2/VLDL2 and n-acetylated glycoproteins, as well as metabolites such as 1-methylhistidine and methionine, thus verifying the relationship between abnormal lipid panels and patients with hypothyroidism. The control samples exhibited elevated levels of phenylalanine, tyrosine, glycerol, alanine, methanol, acetic acid and valine. 

**Figure 2:**
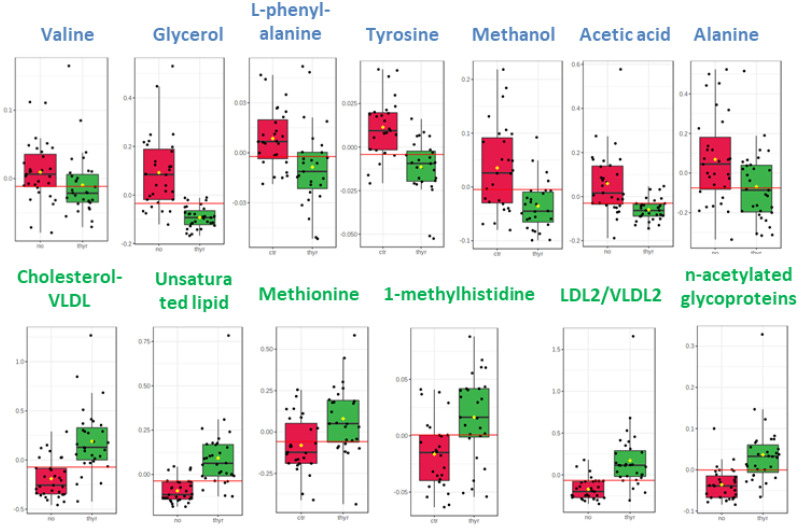
Box plots of discriminant metabolites in serum. (1. Valine, 2. Glycerol, 3. L-phenylalanine, 4. Tyrosine, 5. Methanol, 6. Acetic acid, 7. Alanine, 8. Cholesterol-VLDL, 9. Unsaturated lipid, 10. Methionine, 11. Methylhistidine, 12, LDL2/VLDL2, 13. n-acetylated glycoproteins).

The distinctive metabolic signatures from this comparison are displayed in corresponding box plots (Figure 2).

The circulatory metabolome was dominated by signals from lipids and lipoproteins. There is sufficient documentation that lipid status is affected by thyroid disorder since THs modulate cholesterol’s synthesis, mobilization and breakdown [34,35]. Dyslipidemia is a significant comorbidity of thyroid disease, with multiple long-term negative effects [36]. The differences in the levels of the essential amino acids methyl histidine and phenylalanine and in the levels of the non-essential amino acids alanine and tyrosine may simply indicate either a differential rate to meet the requirements for elementary building blocks or a comparative under-utilization in gluco-neogenesis.

N-acetyl signals may arise from acute-phase glycoproteins known to reflect inflammatory statuses [37]. THs modulate inflammation processes and are implicated in placental development and disease. The ratio of free T3:free T4 is positively associated with the adiposity-related inflammation markers interleukin-6 and C-reactive protein [38]. Recent studies reported that leptin signaling, mediated by the JAK (Janus kinase)/STAT (signal transducer and activator of transcription) pathway, plays a vital role in maintaining TRH, TSH and thyroid hormone expressions [38]. Obesity is characterized by enlarged adipose cells and chronic inflammation and is critically linked to hypothyroidism [38]. Additionally, thyroid autoimmunity is highly related to the regulation of inflammasome-related cytokines in obesity [38].

Hypothyroid patients have increased serum LDL cholesterol levels and a reduced LDL uptake [39]. Thyroid hormones regulate the serum levels of cholesterol by stimulating cholesterol biosynthesis, exports (primarily as VLDL and LDL), reverse transports from peripheral tissues, hepatic reuptakes via LDL receptors (LDLRs) and conversions into bile acids in the liver. Data suggest that even slightly low levels of thyroid hormones can cause a spike in cholesterol.

In line with our results, Wu et al. [40] implemented a serum metabonomic profiling method based on ultra-performance liquid chromatography/time-of-flight mass spectrometry (UHPLC/TOF-MS) in order to investigate the metabolic changes in hypothyroid rats and identified a higher trend in the levels of L-phenylalanine, L-tyrosine and methionine. Moreover, the content of 1-methylhistidine was also changed in the experimental rat models of hypothyroidism, but contrary to our results, was decreased in the hypothyroid samples, thus bearing potentially serious repercussions for the functioning of the rats’ skeletal muscles [41,42].

Aminoacid levels, such as alanine, valine, phenyl alanine and tyrosine, have been found to be low in hypothyroid female human samples, but this may have been partly attributed to their nutrition, since these women followed a meat-rich diet (Figure S4).

Alanine has been correlated with a higher blood pressure, high energy intake, high cholesterol levels and increased body mass index. Alanine plays a key role in the glucose–alanine cycle between the tissues and liver, enabling pyruvate and glutamate to be removed from the muscle and be reformed in the liver [43].

Glycerol is important for the lipid metabolism and mitochondrial energy production based on lipids. Glycerol and free fatty acids (FFA) are produced from the lipolysis of triglycerides stored in adipose tissue. Glycerol formed during the hydrolysis of glycerides is not reutilized in the adipose tissue but is either oxidized in or transformed into glucose in glycerokinase-containing tissues (kidneys, intestinal mucosa and liver) [44].

Our study’s decrease in acetate in the group with thyroid dysfunction might be attributed to higher levels of non-essential fatty acids, implying a lower insulin sensitivity in the adipose tissue, allowing for a weaker suppression of lipolysis.

### 2.2. Colostrum Metabolome

The colostrum samples from the same mothers that provided the serum specimens were also analyzed to contribute to the results interpretation. A typical standard ^1^H NMR spectrum of colostrum with the identified metabolites acquired with the NOESYPRESAT pulse sequence is depicted in Figure S5.

In line with the investigation of the serum samples, the first PCA was implemented on the colostrum samples after the outlier exclusion to provide an overview and reveal the trends of the samples’ clustering related to thyroid disorder (Figure S6).

**Figure 3:**
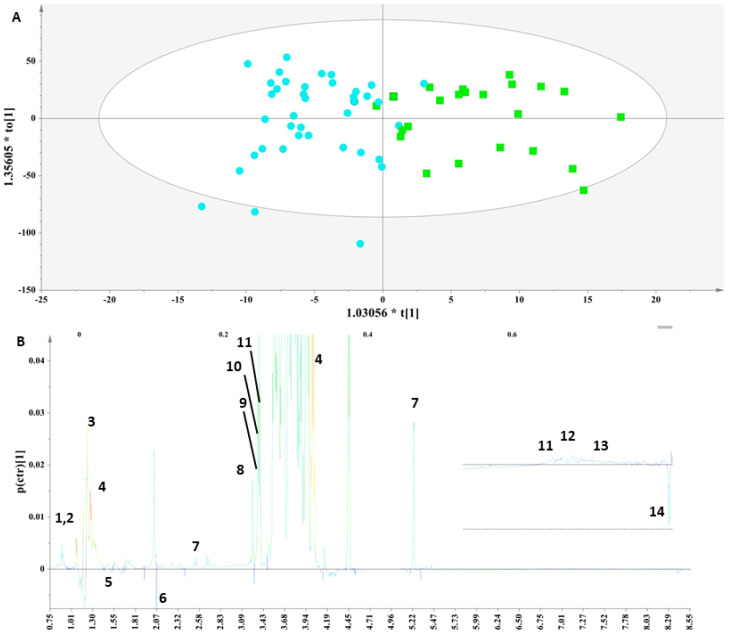
(**A**) OPLS-DA model; A = 1 + 1, N = 62; R^2^X(cum)= 0.85 and R^2^Y(cum) = 0.62; Q^2^(cum) = 0.49. Green squares = samples with thyroid disorder and blue circles= control samples. (**B**) S-line plot (1: leucine (2-amino-4-methylpentanoic acid), 2: isoleucine (2-amino-3-methylpentanoic acid), 3: lactate (2-hydroxypropanoic acid), 4: threonine (2-amino-3-hydroxybutanoic acid), 5: alanine (2-aminopropanoic acid), 6: N-acetylmoieties, 7: lactose, 8: choline ((2-hydroxyethyl)trimethylazanium), 9: o-phosphocholine ([2-(trimethylazaniumyl)ethoxy]phosphonic acid), 10: glycerophosphocholine ((2-{[(2R)-2,3-dihydroxypropyl phosphono]oxy}ethyl)trimethylazanium), 11: t-methyl histidine (3-(1H-imidazol-5-yl)-2-(methylamino)propanoic acid), 12: tyrosine (2-amino-3-(4-hydroxyphenyl)propanoic acid), 13: phenylalanine (2-amino-3-phenylpropanoic acid) and 14: formic acid).

We implemented a supervised analysis on the samples from the PCA model in Figure S6 to examine whether during the early stages of lactation, the nutritional composition of human milk is affected due to thyroid disorder. Toward this aim, the PCA workset was used as a training set to produce the first OPLS-DA model (Figure 3A), and then a prediction set of 10 samples and a validation set of 16 samples comprised the final OPLS-DA model (Figure S7).

Specifically, an OPLS-DA model (Figure 3A) was extracted that correctly classified 79.17% of the colostrum samples. This model clearly discriminated the samples along the first component and indicated that the colostrum samples from the mothers with thyroid disorder were characterized by significantly higher levels of threonine, lactate, choline, choline phosphate and glycerophosphocholine, as depicted in the corresponding S-line plot (Figure 3B). The lactose levels were higher in this group but not significantly. Moreover, although increased levels of alanine, citric acid and formic acid were noticed in the control samples, these differences were not significant.

**Figure 4:**
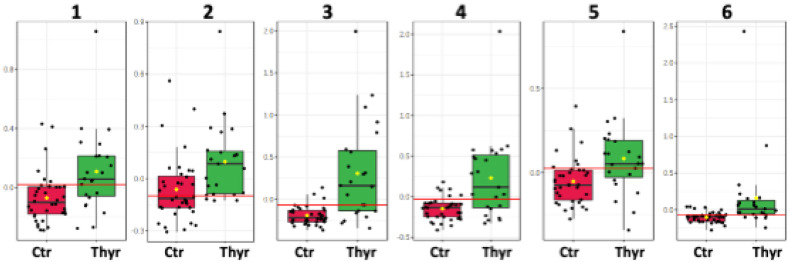
Box plots of the discriminant metabolites in colostrum. (1. Glycero-phosphocholine, 2. o-phosphocholine, 3. Fucosyl moieties, 4. Lactate, 5. Choline, 6. Threonine).

The metabolic trends of this comparison are framed in box plots as presented in the Figure 4.

Finally, the use of validation steps (*p* value< 0.05, permutation testing and ROC curves), as described in the materials and methods section, confirmed that the results of all the OPLS-DA models in each substrate were unbiased and reliable, as described in Figure S8. The metabolites in both the biofluids highlighted by the S-line plot with *p* values < 0.005 are presented in Table S2.

Consistent with the investigated-serum sample pool, the levels of membrane and lipid metabolites, (i.e., LDL/VLDL, choline, glycerophosphocholine, etc.), were found to be significantly increased similar to the samples with thyroid disorder of both biofluids.

A recent ^1^H NMR-based metabolomics study by Zhao et al. [22] monitored perturbations of metabolites on the cord blood of 18 pregnant women with GHT and 18 non-hypothyroidism (NHT) control metabolites and observed an increase in the levels of o-phosphocholine on downregulated samples with GHT.

Choline is an essential constituent of membrane phospholipids in great demand in the developing brain and liver. An adequate supply of choline is thought to be particularly important during fetal development [45]. The need for choline is also likely to be increased during pregnancy and lactation since large amounts of choline must be delivered to the fetus across the placenta and transferred from maternal circulation to breast milk [46].

In our study, fruit and vegetable intake may have affected the total choline and individual choline forms present among the participants, so people on vegetarian or plant-based diets may exhibit increased choline concentrations (Figure S9) [47].

Lactose is the most abundant component of human milk and is often measured to reflect carbohydrate energy contents. Human-milk lactose has been associated with infant growth in observational and simulation studies [48,49].Citrate plays a central role as an intermediate in the cell-energy metabolism and the tricarboxylic acid cycle [50]. Furthermore, citric acid mediates iron’s absorption from human-milk fractions [51].

Other studies comparing the human milk of patients with thyroid disorder to that of healthy subjects have reported changes in the protein, fat and lactose compositions of the human milk, evident only after the first week postpartum [30]. Such alterations in the composition of human milk can be explained since thyroid hormones are implicated in the pathways that mediate the tissue metabolism of carbohydrates, lipids and proteins [52]. Moreover, it should be noted that these hormones are galactopoietic and prioritize the metabolic activity of the mammary gland during both gestation and lactation [53].

In this context, our results of both biofluids are in line with an increasing body of evidence from recent studies showing that lower serum fT4 and/or higher TSH levels can be related, in euthyroid pregnant women, to a less-favourable metabolic phenotype. As pregnancy progresses, there is an overt increase in adipose tissue, insulin resistance and immunosuppression. Even with a prompt treatment initiation, a euthyroid status will not be achieved if there is not a proper follow-up and dose adjustment of T4 since LT4 doses often need to be increased by gestational week 4–6 and may require a 30–50% increment in the dose. Recent studies have highlighted a relationship between markers of metabolic dysfunction and the occurrence of physiological variations in thyroid function. Although these associations are well documented for euthyroid adults, similar reports for pregnant women are lacking [54].

### 2.3. Receiver-Operating Characteristic (ROC) Curve Analysis for Metabolite Markers

We performed an ROC analysis after the delineation in each specimen of a panel of significant metabolites to assess a quantitative measure for discriminatory potential. 

Specifically, an ROC curve was computed using MetaboAnalyst for each one of the significant metabolites, as discussed above (Figures S10 and S11), in order to elucidate the putativemetabolite markers that express the impact of thyroid disorder on both biofluids and to avoid false selections.

In fact, for the serum samples, glycerol and unsaturated lipids exhibited AUROCs > 0.9, while phenylalanine, methionine, cholesterol VLDL, tyrosine, acetic acid, n-acetylated glycoproteins, lipids (VLDL/LDL) and 1-methylhistidine exhibited AUROCs > 0.7. These should be considered the most fitting metabolite markers of thyroid disorder in the serum samples (Figure S10).

Moreover, for the colostrum samples, 2-fucosyl lactose, threonine, o-phosphocholine, glycerophosphocholine, choline and lactate exhibited AUROCs > 0.7. These should be considered the most fitting metabolite markers of thyroid disorder in the colostrum samples (Figure S11).

### 2.4. MetabolitePathway Analysis

**Figure 5:**
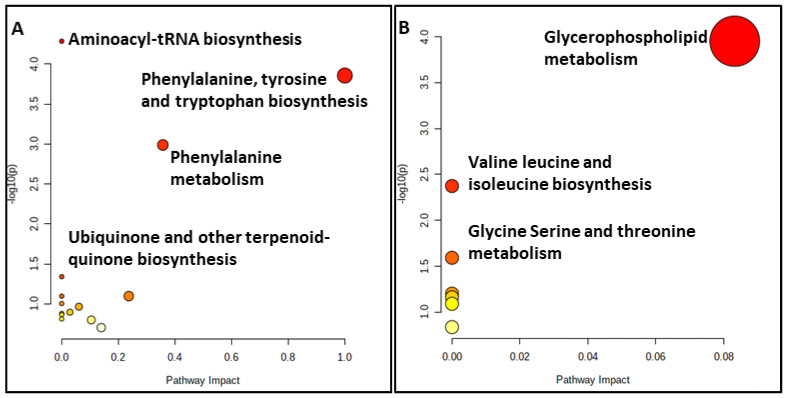
Summary plots for over-representation analysis of the (**A**) serum and (**B**) colostrum substrates.

A metabolite pathway analysis using MetaboAnalyst 5.0 [55] was performed to identify the most relevant metabolic pathways reflected in the serum and colostrum based on the identified metabolites with AUROCs > 0.7. The results of the pathway analysis for both substrates are depicted in Figure 5.

Our results for the serum substrate revealed that the primary disturbed statistically significant pathways (*p* < 0.05), in response to hypothyroidism, were aminoacyl-tRNA biosynthesis, phenylalanine, tyrosine and tryptophan biosynthesis, phenylalanine metabolism and ubiquinone and other terpenoid-quinone biosyntheses (Table S3). The relationship between the ubiquinone metabolism and thyroxine status has been reported since the late 1960s, with higher levels of thyroxine leading to higher ubiquinone concentrations and accumulations in the liver due to an increased biosynthesis and to a partly decreased catabolism [56]. Specifically, the pathways of importance containing at least two compounds involve phenylalanine, tyrosine and tryptophan biosynthesis, aminoacyl-tRNA biosynthesis and the phenylalanine metabolism.

However, the statistically significant pathways (*p* < 0.05) for the colostrum substrate are glycerophospholipid metabolism, glycine, serine and threonine metabolism and valine, leucine and isoleucine biosynthesis (Table S4). Only the glycerophospholipid metabolism and glycine, serine and threonine metabolism contained at least two metabolites of those subjected to the pathway analysis.

Thyroid hormones are known to affect various metabolic pathways. The primary actions include an increase in the basal metabolic rate and the coordination of normal growth and development. Maternal serum lipid concentrations increase as pregnancy progresses. The TSH early in pregnancy contributes to the development of the fetal central nervous system (CNS) and the coordination of the fetal metabolic processes. The dysregulation of glucose and lipid metabolism has been linked to several metabolic diseases, such as gestational diabetes mellitus (GDM), and therefore, maternal thyroid disease may potentially contribute to the diversion of fetal metabolic processes. In particular, hypothyroidism has been associated with a decreased metabolic rate as well as a decrease in various metabolic procedures, including proteolysis, protein synthesis, lipogenesis, lipolysis, gluconeogenesis and glycogenolysis. 

### 2.5. Metabolite–Disease Relation Network Analysis

**Figure 6:**
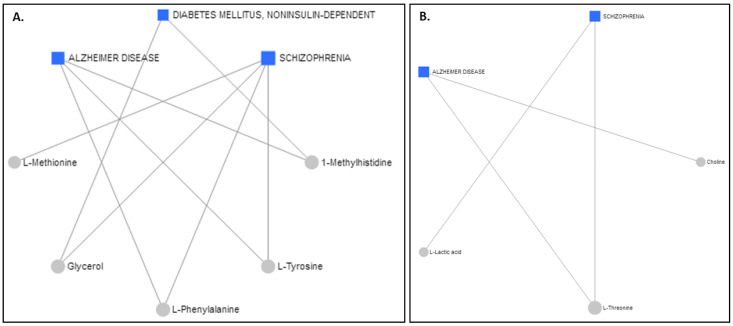
Schematic diagram illustrating disease-related metabolites and relevant pathways based on the metabolite data from (**A**) serum and (**B**) colostrum.

The Network Explorer analysis module of MetaboAnalyst 5.0 [55] was used to explore the metabolic pathways and relate the interactions among the metabolites to diseases. After the incorporation of the metabolites exhibiting AUROCs > 0.7, a metabolite–disease interaction network was extracted, identifying metabolite–disease connections that cross pathway boundaries. The results for the serum are displayed in Figure 6A and the results for the colostrum are in Figure 6B. Interestingly, the two different biofluids had the same advent outcomes. Alzheimer’s disease and schizophrenia were highlighted as potential disease outcomes for both the serum and the colostrum, whereas diabetes mellitus was suggested for only the serum samples.

Data in the literature suggest that exposure to thyroid disorder during pregnancy may incur health issues related to mental disorders, cognitive impairments and metabolic diseases, such as diabetes mellitus [57]. Our findings corroborate this concept and further enable us to document, for the first time, the fact that maternal thyroid-disorder patterns are reflected in the compositions of human serum and milk. It is unclear whether the alterations in thyroid function represent transient or continuous exposures and whether similar metabolic markers would be found later in the maternal life. Furthermore, the respective differences might be attributed to different hypothyroidism etiologies and pre pregnancy thyroid statuses, treatment initiations, durations and doses as well as indices indicative of metabolic syndrome in our population.

The metabolic profile of the samples with thyroid disorder was dominated by signals from lipids and lipoproteins, implying the occurrence of dyslipidemia, a major risk factor for adverse cardiovascular events. Previous studies revealed molecular mechanisms for the association between hypercholesterolemia and hypothyroidism [58]. A pregnant female with subclinical hypothyroidism (SH) that had a normal thyroid status prior to theirpregnancy will not present dyslipidemia in the beginning of their pregnancy. Moreover, an altered BMI during their pregnancy could possibly be implicated in thyroid dysfunction. The secretion of adiponectin is shown to be upregulated by thyroid hormones in human adipose tissue [54], thus regulating the energy metabolism in pregnancy. In addition, similar associations were found for thyroid hormones and insulin resistance, connecting thyroid function with the glucose metabolism in pregnancy, even in euthyroid women.

Interestingly, one of the biological mechanisms underlying the relationship between thyroid dysfunction and dementia was suggested to be associated with cardiac vascular disease, which can contribute to cognitive impairments later in life [57,59]. Experimental studies reported that thyroid hormones induce changes in the amyloid precursor processing or deposition of amyloid-β, the major component of the brain amyloid deposits found in Alzheimer disease (AD) [60,61,62], thus pinpointing a strong role of thyroid hormones in the etiology of AD [62].Not all studies, however, concur with this relationship since their results have shown either no association [63] or no concordance with an association of hypothyroidism with AD [64]. Yet, despite the literature on this subject, it remains unclear whether thyroid dysfunction results from or contributes to Alzheimer’s pathology. Our results support the latter.

Other studies have revealed a high prevalence of thyroid dysfunction in patients with schizophrenia [65], thus providing insight into the implications of thyroid hormone homeostasis in the fine-tuning of crucial brain networks. Maternal hypothyroxinemia alters fetal brain development, possibly linking the condition to early neurodevelopmental disorders and neurocognitive deficits [66] that primarily set the origin for the later development of psychiatric diseases, such as schizophrenia, in offspring [66].

The two most commonly occurring endocrinopathies during pregnancy are thyroid disease and diabetes, both caused by an autoimmune process. The onset of one autoimmune disease, such as diabetes, in turn increases the risk of developing another autoimmune disease, such as thyroid disease. Additional independent associations were found for thyroid hormones and markers of glucose tolerance and insulin resistance. The impact of thyroid hormones, even within the euthyroid range, on the glucose metabolism in pregnant women cannot be excluded. Subclinical hypothyroidism and isolated hypothyroxinemia in pregnancy have been associated with an increased risk of gestational diabetes. In fact, studies have documented the interdependent relationship between these two conditions and the increased prevalence of thyroid disorders in patients with diabetes mellitus and *vice versa* [67,68]. On one hand, thyroid hormones contribute to the regulation of the carbohydrate metabolism and pancreatic function, and on the other, diabetes affects thyroid function to variable extents [69,70]. 

## 3. Materials and Methods

### 3.1. Ethics Committee

The study protocol was approved by the ethics committee of our teaching hospital, and all recruited mothers signed an informed consent form. 

### 3.2. Patients-Methods

The characteristics of all the women participating in this study are provided in Table S1.

### 3.3. Biofluid Collection

A total of 90 full-term singleton pregnancies were included in our study. Fiftyof them were uncomplicated pregnancies and served as controls and 40 of them were cases complicated by thyroid disease and comprised the study group. The selection of cases of gestational hypothyroidism, as *per* the 2017 Guidelines of the American Thyroid Association [71], was based on laboratory findings during pregnancies; cases with positive personal history for pre-existing thyroid disease were also included. More specifically, our cases had either a first-trimester TSH > 2.5 mIU/mLwith or without the presence of antithyroid autoantibodies (antiTPO) or had positive antiTPO in the context of pre-existing thyroid disease and were already on thyroxine replacement therapy. TSH and antiTPO were measured with immuno-chemiluminescence techniques. 

From each woman, serum and breast milk were collected on the 3rd–4th day postpartum at the same time prior to lunch and after having been fasted for 3 h. After a 45-min incubation at room temperature, blood serum was centrifuged at 6000× *g* for 5 min. One milliliter of the supernatant serum and the same portion of milk were transferred into separate sterile cryovials and were stored at −80 °C. Additionally, in each case, an extended questionnaire was completed via personal interview, which included sociodemographic data, maternal and neonatal anthropometric parameters, medication data, laboratory findings and dietary habits during pregnancy.

### 3.4. Metabolomic Analysis

#### Sample Preparation 

*Colostrum*: Samples were prepared adding 140 μL of phosphate buffer in D_2_O to 400 μL of maternal milk. After centrifugation at 4 °C for 10 min at 10,000× *g*, 50 μL of trimethylsilyl propionate (TSP) was added, as internal standard, to 500 μL of the supernatant and transferred to 5 mm NMR tubes. 

*Serum:* Samples were prepared by adding 140 μL of phosphate buffer in D_2_O to 400 μL of serum. After centrifugation (10,000× *g*, 4 °C, 10 min), 50 μL of sodium maleate was added as internal standard to 500 μL of the supernatant and transferred to 5 mm NMR tubes.

Sodium maleate was chosen as reference standard for serum and AF since it is suitable for CPMG pulse sequence and provides a distinct peak in the ^1^H NMR spectrum [72].

### 3.5. NMR Analysis

All samples were thawed at room temperature for 60 min prior to performing the NMR experiments. All NMR spectra were acquired on a Varian600-MHz NMR spectrometer equipped with a triple resonance probe {HCN}. One-dimensional ^1^H NMR spectra were collected at 25 °C. Receiver gain was kept constant for all acquisitions.

For the colostrum samples, a 1D NOESYPRESAT pulse sequence was applied with 128 transients collected with 64K data points. Relaxation delay was set to 2 s.

For the serum samples, a representative proton Carr–Purcell–Meiboom–Gill nuclear magnetic resonance (^1^H CPMG NMR) pulse sequence was then applied with 128 transients collected with 64K data points. Relaxation delay was set to 6 s. Proton spectra were referenced at the resonance peak of sodium maleate (5.95 ppm).

### 3.6. Data Preprocessing 

All ^1^H NMR spectra were phase and baseline corrected using the softwareMnovaver.10.1. The NMR spectrum of each sample was aligned based on the reference compound’s signal. The ^1^H NMR spectra were reduced into buckets of 0.0001 ppm, and theD_2_O region was removed. The spectra were normalized to the standardized area of the reference compound and converted into ASCII format using the softwareMnovaver.10.1. The ASCII format files were imported into MATLAB (R2006a, MathWorks Inc., 2006, Natick, MA, USA), and all spectra were aligned using the correlation optimized warping (COW) method [73].

### 3.7. Annotation of Metabolites

In particular, Metaboneer, an in-house fully automated metabolite identification platform [74], facilitated the resonance-peak identification of 37 metabolites in serum (Table S5). The identification procedure was also assisted by literature data [16,75]. 

A series of 2D experiments, i.e., gCOSY, zTOCSY, gHMBCad and gHSQCad experiments, were recorded at 25 °C and permitted the assignment of metabolites. Table S6 summarizes the chemical shifts (ppm) of the identified metabolites. The acquisition parameters for gCOSY were: spectral width, (SW) 7225.4 Hz; t1 increments, 256; acquisition time, 0.150 s; number of scans, 128; data points, 1084; receiver gain, 30 and relaxation delay, 1 s. The zTOCSY experiment was performed with: spectral width (SW), 7225.4 Hz; t1 increments, 256; number of scans, 128; acquisition time, 0.283 s; data points, 2048; receiver gain, 30 and relaxation delay, 1 s. The acquisition parameters for gHSQCad were: f2 spectral width (SW), 7225.4 Hz; f1 spectral width (SW), 30165.9 Hz; t1 increments, 256; number of scans, 128; acquisition time, 0.150 s; data points, 1084; receiver gain, 30 and relaxation delay, 1 s. The acquisition parameters for gHMBCad were: f2 spectral width (SW), 7225.4 Hz; f1 spectral width (SW), 36199.1 Hz; t1 increments, 256; number of scans, 128; acquisition time, 0.150 s; data points, 1084; receiver gain, 40 and relaxation delay, 1 s. The interpretation of 2D spectra was performed with the use of the software MestReNova (ver.10.1, Santiago de Compostela, Spain). 

### 3.8. Statistical Analysis 

#### 3.8.1. Postprocessing of Spectral Data

SIMCA-P (version 14.0, Umetrics, Umeå, Sweden) was facilitated. The spectral data were mean-centered Pareto scaled (Par), and the PCA, as well as the OPLS-DA models, were extracted at a confidence level of 95%. The mathematical background and applications of these methods have been extensively discussed [76].

#### 3.8.2. Identification of Important Features 

S-line plots were facilitated to pinpoint the metabolites that contributed to the samples’ discrimination. 

#### 3.8.3. Model Validation 

The quality of models (PCA/OPLS-DA) was described by the goodness-of-fit R^2^ (0 ≤ R^2^ ≤ 1) and the predictive ability Q^2^ (0 ≤ Q^2^ ≤ 1) values. The R^2^ explained the variation, thus constituting a quantitative measure of how well the data of the training set was mathematically reproduced. The overall predictive ability of the model was assessed by using the cumulative Q^2^, representing the fraction of the variation of Y that could be predicted by the model, which was extracted according to the internal cross-validation default method of the software SIMCA-P. Q^2^ is considered a *de facto* default diagnostic parameter for validating OPLS-DA models in metabolomics. In particular, the difference between the goodness of fit and the predictive ability remained always lower than 0.3 (R^2^X(cum) − Q^2^(cum) < 0.3), and the goodness of fit never equaled one (R^2^X(cum) ≠ 1). Therefore, since the extracted models abided by these rules, their robustness and predictive response were enhanced and over-fitting was effaced.

Regression models were validated using cross-validation analysis of variance (CV-ANOVA) with a *p* value < 0.05. Furthermore, permutation tests were employed (999 permutations) to evaluate whether the specific classification of two classes in a model were significantly better than any other models obtained by randomly permuting the original group’s attribution. An additional measure of PLS-DA model validity included the extraction of receiver-operating characteristic (ROC) curves to assess the ability of the PLS latent variable Tpred to correctly classify the test set. A marker explained a low, fair and superior diagnostic accuracy when the area under the ROC (AUROC) curve reached values of 0.5 < AUC < 0.7, 0.7 < AUC < 0.9 and AUC > 0.9, respectively. The area under the ROC (AUROC) was calculated. A perfect discrimination corresponded to an AUROC equal to 1. 

#### 3.8.4. Metabolic Pathways

The online software MetaboAnalyst (5.0, Alberta, AB, Canada) [55] was utilized for biomarker discovery, classification and pathway mapping of metabolites exhibiting AUROCs > 0.7 to enable exploration of disease-related metabolites and pinpoint the most relevant pathways.

The network explorer analysis module capitalizes on the general concept of knowledge-driven network by analyzing each set of -omics data then mapping the significant metabolite features in the forms of networks in order to uncover meaningful links among them as well as their associations with disease phenotypes.

## 4. Conclusions

Overall, the study highlights the efficiency of NMR metabolomics coupled with multivariate pattern recognition analysis in identifying novel diagnostic and prognostic biomarkers of thyroid statuses and metabolomics’ potential to improve clinical management. This study encompassed a competent sample pool of two biofluids with a prospective and systematic collection of maternal serum and colostrum samples to document thyroid treatments and the comorbidities presented throughout the respective pregnancies. Our results of the metabolic profiles of maternal serum and colostrum clearly showed that women with thyroid disorder, despite replacement therapy, exhibit a different metabolic phenotype, which distinguished them from controls. The AUC of the biomarker panel containing 10 and 4 metabolites, respectively, for the circulatory metabolome and the colostrum models indicated a high predictive ability, and these changes were also found to be in good agreement with the disease pathophysiology. It is unclear whether the alterations in thyroid function represented a transient or continuous exposure and whether similar metabolic markers would have been found later in the mothers’ lives. Furthermore, the respective differences might be attributed to different hypothyroidism etiologies and prepregnancy thyroid statuses, treatment initiations, durations and dosages, as well as indices indicative of metabolic syndrome in our population. Under this light, the correlation of metabolic data allowed the visualization of the affected metabolic pathways that are implicated in critical periods for offspring development. On one hand, the serum analysis of our group of women with thyroid dysfunction in the first trimester of gestation reflected fetal development, and on the other hand, the colostrum analysis reflected the rapid growth and optimal CNS development of breastfeeding infants in the first 6 months of life. In this respect, it would be safe to assume that metabolic mechanisms altered due to gestational hypothyroidism can set the basis for later occurrences of adult diseases that affect cognition, neurodevelopment and metabolism, namely Alzheimer disease, schizophrenia and diabetes mellitus. In particular, the altered metabolic phenotype of maternal milk in the study population is of special interest considering the global promotion of breastfeeding. Further studies on well-defined groups of women with gestational hypothyroidism are expected to elucidate respective prognostic and diagnostic biomarkers. Our findings emphasize the need to follow-up with women adequately after initiating treatments.

## Data Availability

The data presented in this study are available on request from the corresponding author. The data are not publicly available due to ethical restrictions.

## Supplementary Materials

The following supporting information can be downloaded at: https://www.mdpi.com/article/10.3390/ijms23084248/s1.
