# Supplementary material for: Uncontrolled Thyroid during Pregnancy Alters the Circulative and Exerted Metabolome
Source: Int J Mol Sci. 2022 Apr 12;23(8):4248. doi: 10.3390/ijms23084248 (PMC9029102; doi:10.3390/ijms23084248)
Supplement: Supplementary file 1 [file ijms-23-04248-s001.zip › ijms-1639373-supplementary.pdf]

Supplementary Material

Figure S1. A characteristic NMR spectrum acquired with the CPMG pulse sequence of the serum blood is displayed in with annotations on the identified metabolites (1. Valine, ((2S)-2-amino-3-methylbutanoic acid), 2.Lactate ((2S)-2-hydroxypropanoic acid), 3.Glucose, ((2R,3R,4S,5S,6R)-6-(hydroxymethyl)oxane-2,3,4,5-tetrol), 4.Leucine ((2S)-2-amino-4-methylpentanoic acid), 5.Isoleucine ((2S,3S)-2-amino-3-methylpentanoic acid), 6.Alanine ((2S)-2-aminopropanoic acid), 7.Creatine (2-(N-methylcarbamimidamido)acetic acid), 8.1-Methyl histidine (1-Methyl histidine), 9.Tyrosine ((2S)-2-amino-3-(4-hydroxyphenyl)propanoic acid), 10.Formic acid (Formic acid), 11.Phenyl alanine Phenylalanine ((2S)-2-amino-3-phenylpropanoic acid), 12.n-Acetylglutamine ((2S)-4-carbamoyl-2-acetamidobutanoic acid), 13. Glutamine ((2S)-2-amino-4-carbamoylbutanoic acid), 14. Acetoacetate (3-oxobutanoic acid), 15. Glutamic acid ((2S)-2-aminopentanedioic acid), 16.Acetic acid (Acetic acid), 17.Acetone (propan-2-one), 18.Citric acid (2-hydroxypropane-1,2,3-tricarboxylic acid), 19.Choline ((2-hydroxyethyl)trimethylazanium), 20.Creatinine (2-imino-1-methylimidazolidin-4-one), 21.Phosphorylcholine ([2-(trimethylazaniumyl)ethoxy]phosphonic acid), 22.Methylamine (methanamine), 23. Dimethylamine (Dimethylamine), 24.L-Aspartic acid ((2S)-2-aminobutanedioic acid), 25.L-Asparagine ((2S)-2-amino-3-carbamoylpropanoic acid), 26.3-Hydroxybutyric acid ((3R)-3-hydroxybutanoic acid), 27. Glycine (2-aminoacetic acid), 28.Betaine ((carboxymethyl)trimethylazanium), 29.2-Hydroxybutyric acid ((2S)-2-hydroxybutanoic acid), 30.Isobutyric acid (2-methylpropanoic acid), 31.L-Threonine ((2S,3R)-2-amino-3-hydroxybutanoic acid), 32. D-Lysine, ((2R)-2,6-diaminohexanoic acid))

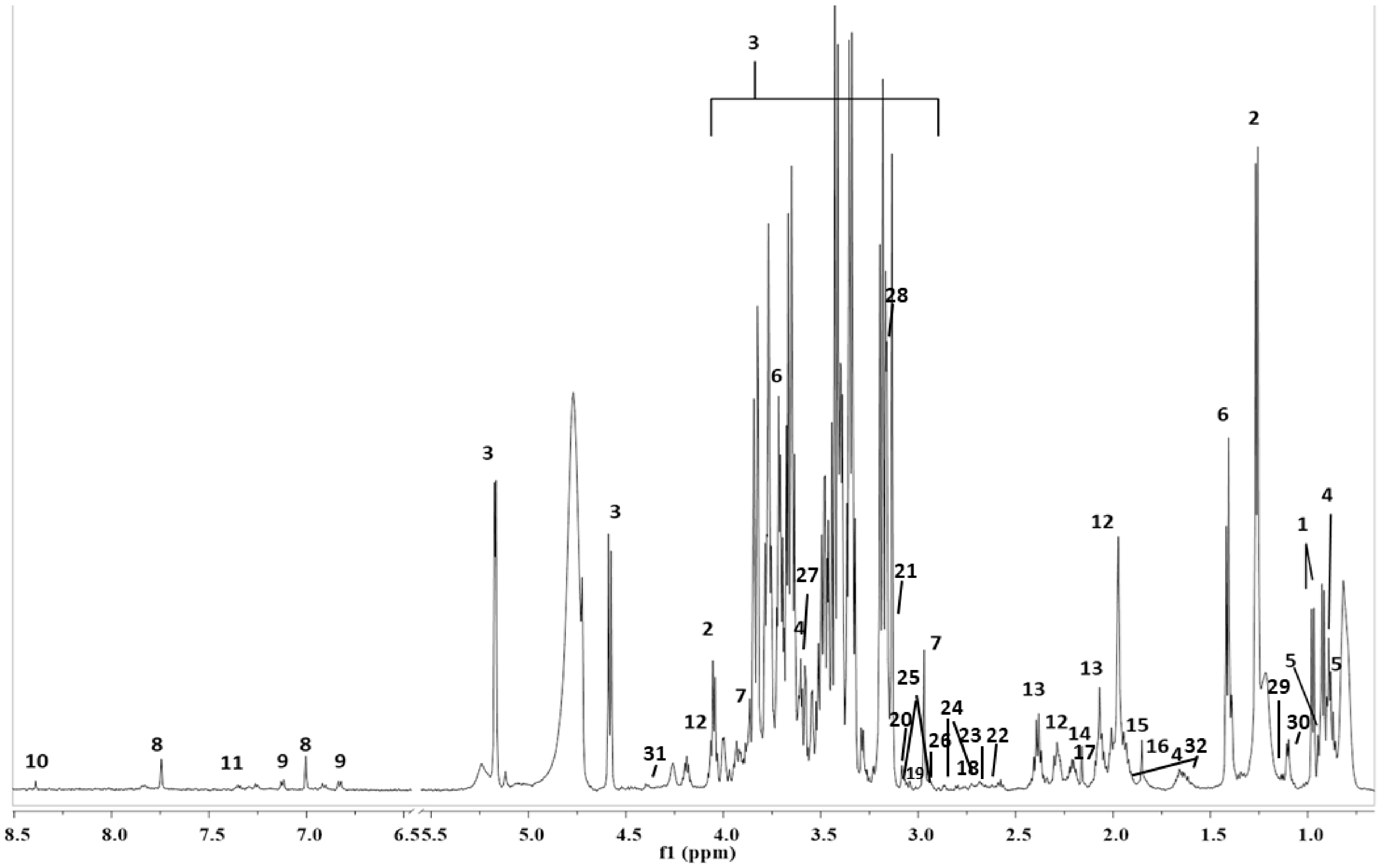

Figure S2. PCA model, N=81; R^2X(cum)= 0.63; Q^2(cum)=0.58. Green squares =samples with thyroid disorder & blue circles = Control samples.

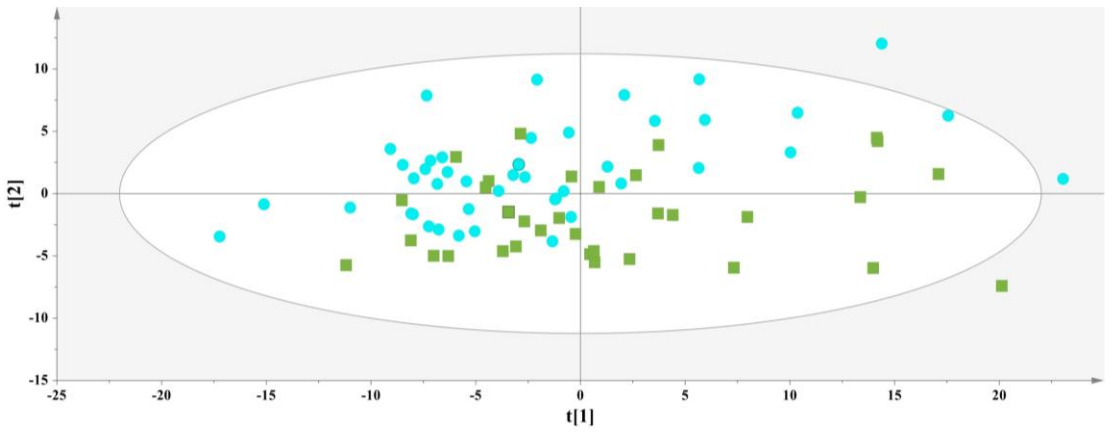

Figure S3. OPLS-DA model, A=1+1; N=76; R^2X(cum)= 0.59; R^2Y(cum)=0.45; Q^2(cum)=0.33. Green squares=samples with thyroid disorder & blue circles= Control samples.

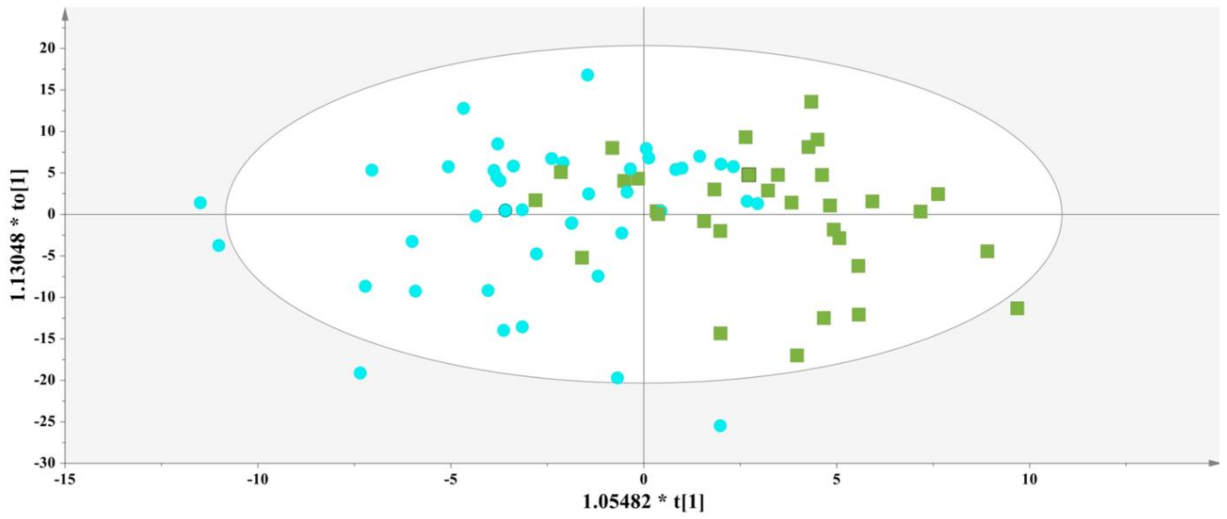

Figure S4. Box plots regarding the meat intake of the participants

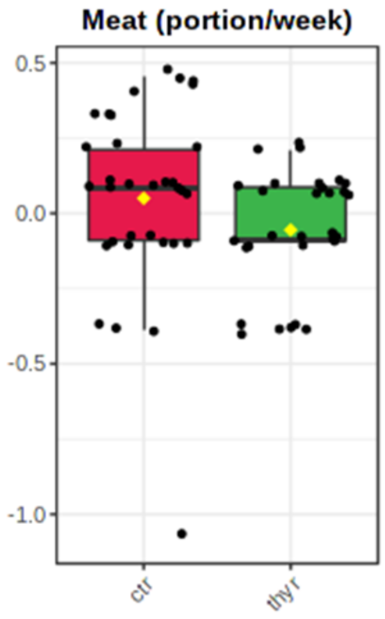

Figure S5. A typical is depicted standard ^{1}H NMR spectrum of colostrum with the NOESYPRESAT sequence (1. Leucine ((2S)-2-amino-4-methylpentanoic acid), 2. Isoleucine ((2S,3S)-2-amino-3-methylpentanoic acid), 3. fucosyl moieties , 4. Lactate ((2S)-2-hydroxypropanoic acid), 5. Threonine ((2S,3R)-2-amino-3-hydroxybutanoic acid, 6. Alanine ((2S)-2-aminopropanoic acid), 7. Ch3GlcNaC (Ch3GlcNaC), 8. citric acid (2-hydroxypropane-1,2,3-tricarboxylic acid), 9. Choline ((2-hydroxyethyl)trimethylazanium), 10. o-phosphocholine ([2-(trimethylazaniumyl)ethoxy]phosphonic acid), 11. glycerophosphocholine((2-{[(2R)-2,3-dihydroxypropyl phosphono]oxy}ethyl)trimethylazanium), 12. Cytidine (4-amino-1-[(2R,3R,4S,5R)-3,4-dihydroxy-5-(hydroxymethyl)oxolan-2-yl]-1,2-dihydropyrimidin-2-one), 13. Lactose ((2R,3R,4S,5R,6S)-2-(hydroxymethyl)-6-{[(2R,3S,4R,5R,6R)-4,5,6-trihydroxy-2-(hydroxymethyl) oxan-3-yl]oxy}oxane-3,4,5-triol), 14. Tyrosine ((2S)-2-amino-3-(4-hydroxyphenyl)propanoic acid), 15. L-phenyl alanine ((2S)-2-amino-3-phenylpropanoic acid), 16. τ-merhyl histidine (τ-merhyl histidine), 17. formic acid (formic acid))

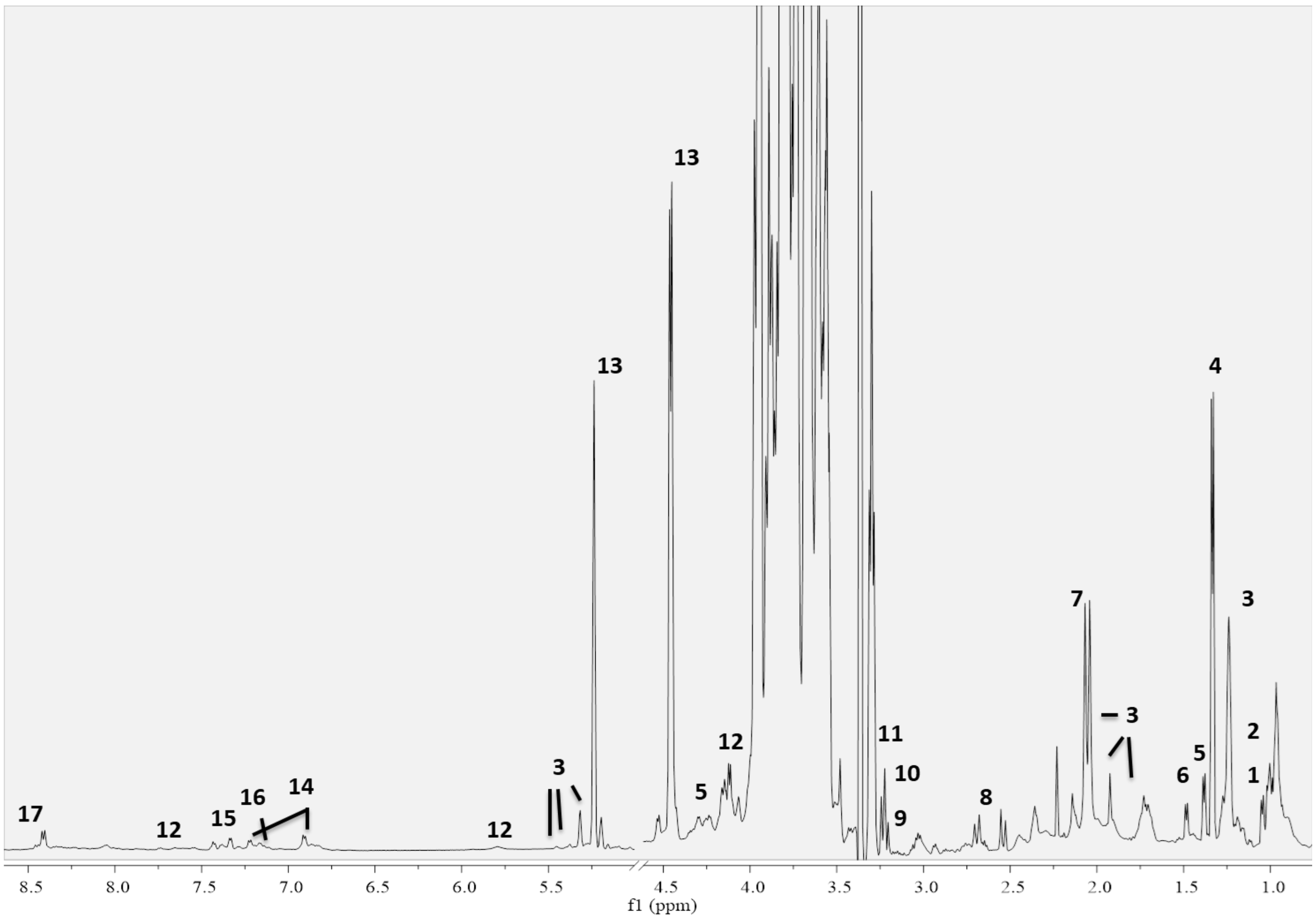

Figure S6. PCA model, N=84; R^2X(cum)= 0.44; Q^2(cum)=0.39. Green squares =samples with thyroid disorder & blue circles = Control samples.

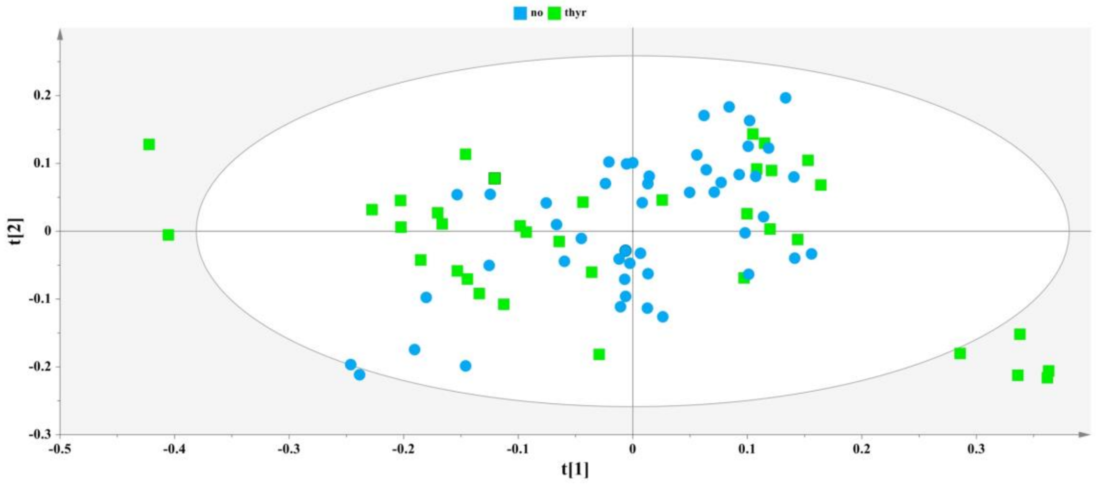

Figure S7. OPLS-DA model, A=1+1; N=78; R^2X(cum)= 0.29; R^2Y(cum)=0.65; Q^2(cum)=0.49. Green squares=samples with thyroid disorder & blue circles= Control samples.

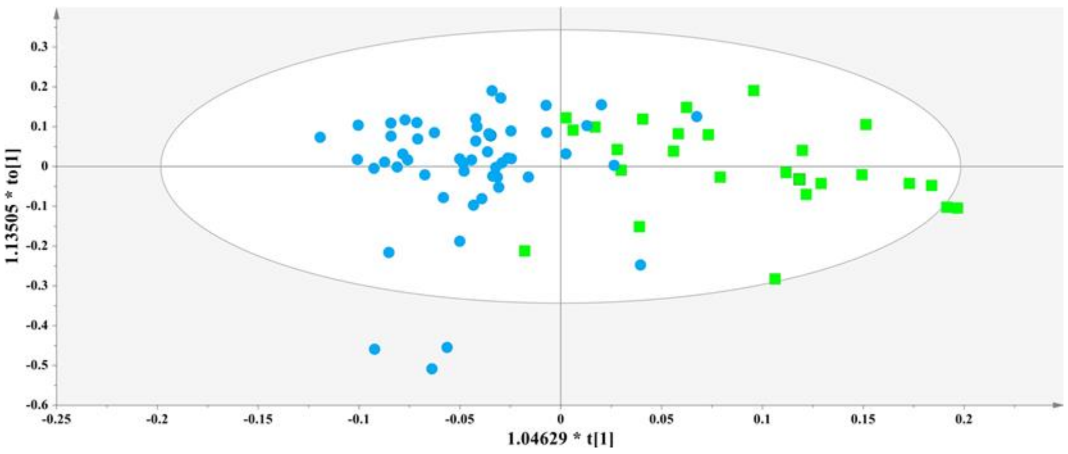

Figure S8. (A) ROC graph AUC (no)= 0.72 and AUC(thyr)=0.85 (B) permutation testing for the OPLS-DA model in Figure 1 and (C) ROC graph AUC (no)= 0.72 and AUC(thyr)=0.79 (D) permutation testing for the OPLS-DA model Figure 3, respectively; (E) ROC graph AUC (no)= 0.90 and AUC(thyr)=0.90 (F) permutation testing for the OPLS-DA model in Figure S3 and (G) ROC graph AUC (no)= 0.78 and AUC(thyr)=0.96 (H) permutation testing for the OPLS-DA model Figure S7, respectively.

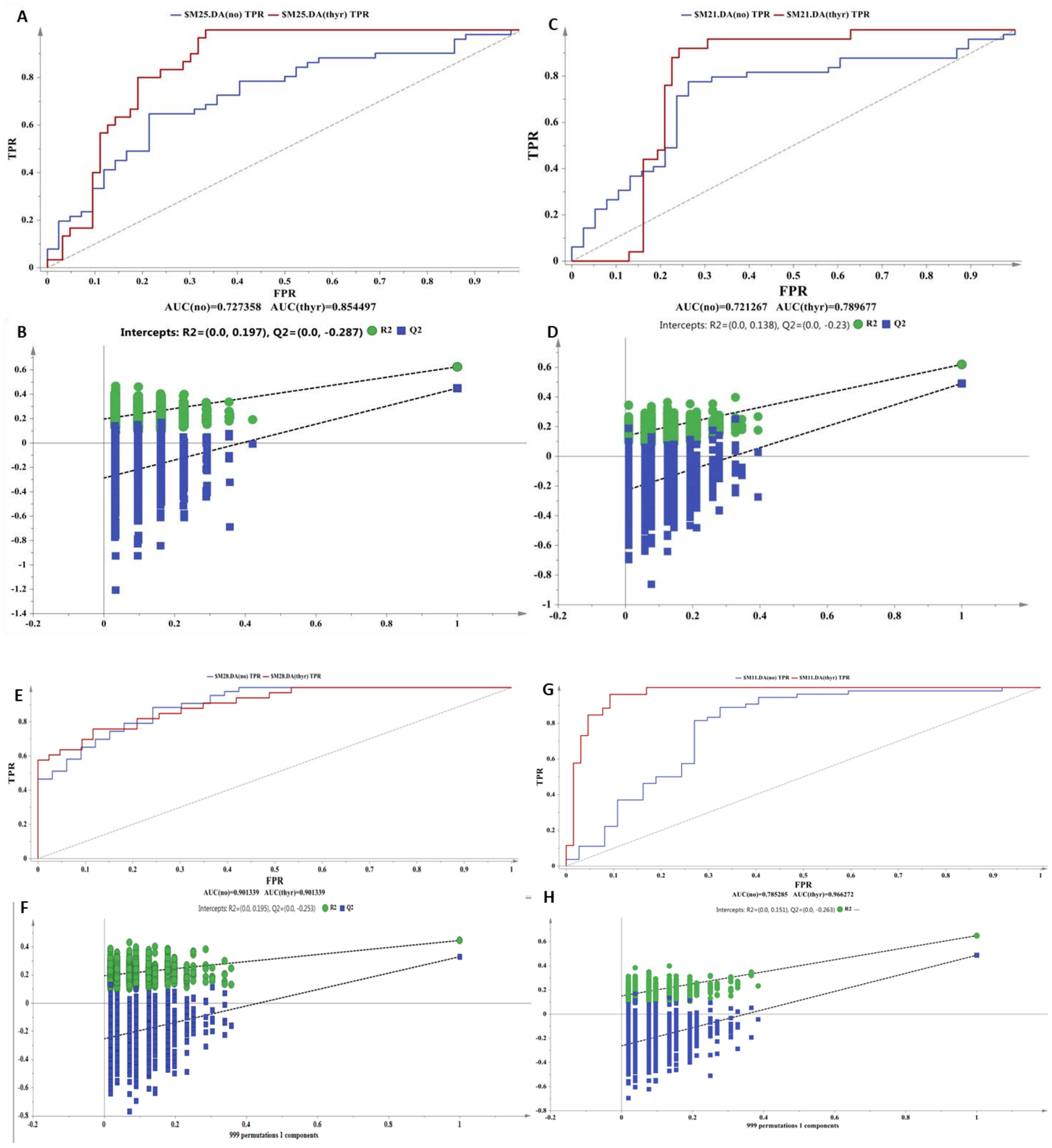

Figure S9 Box plots for fruit intake of the participants

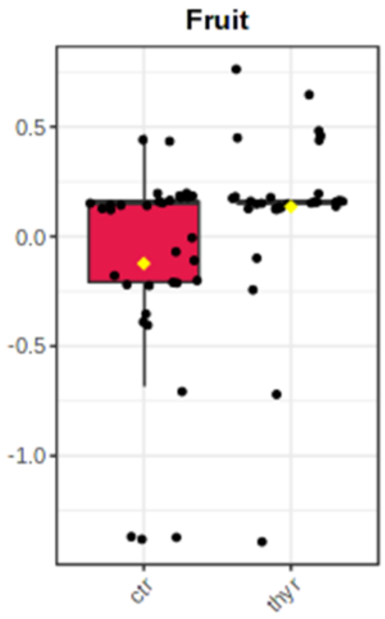

Figure S10 ROC graph with the AUC value for discriminant metabolites in the serum substrate. 1. Valine ((2S)-2-amino-3-methylbutanoic acid), 2. Glycerol (propane-1,2,3-triol), 3. L-phenyl alanine (2S)-2-amino-3-phenylpropanoic acid), 4. Tyrosine ((2S)-2-amino-3-(4-hydroxyphenyl)propanoic acid), 5. Alanine ((2S)-2-aminopropanoic acid), 6. Methanol, 7. Acetic acid, 8. Cholesterol-VLDL ((3β)-cholest-5-en-3-ol-VLDL), 9. Unsaturated lipid, 10. 1-Methyl histidine, 11. methionine (2-amino-4-(methylthio)butanoic acid), 12.LDL2/VLDL2 , 13. n-acetylated glycoproteins

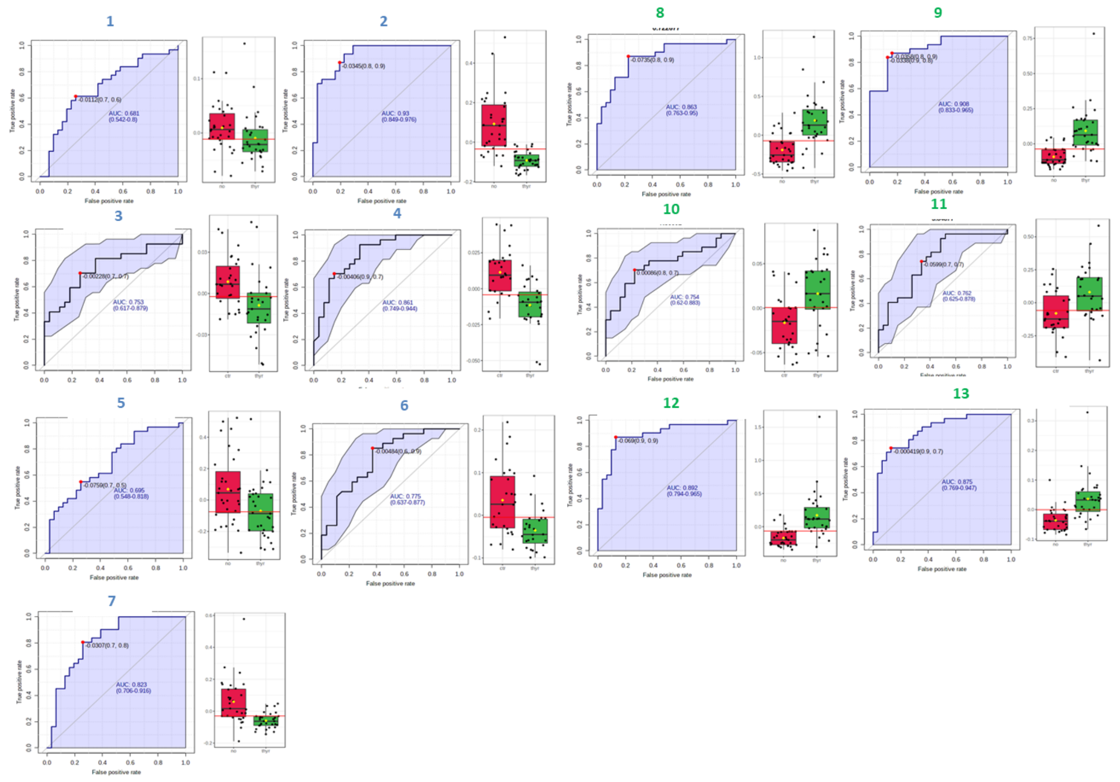

Figure S11 ROC graph with AUC for discriminant metabolites in the colostrum substrate. 1. Fucosyl moieties, 2. Threonine ((2S,3R)-2-amino-3-hydroxybutanoic acid), 3. o-phosphocholine (2-(trimethylazaniumyl) ethoxy]phosphonic acid), 4. Lactate ((2S)-2-hydroxypropanoic acid), 5. Glycerol-phosphocholine ((2-{[(2R)-2,3-dihydroxypropyl phosphono]oxy}ethyl)trimethylazanium), 6. Choline (2-hydroxyethyl)trimethylazanium)

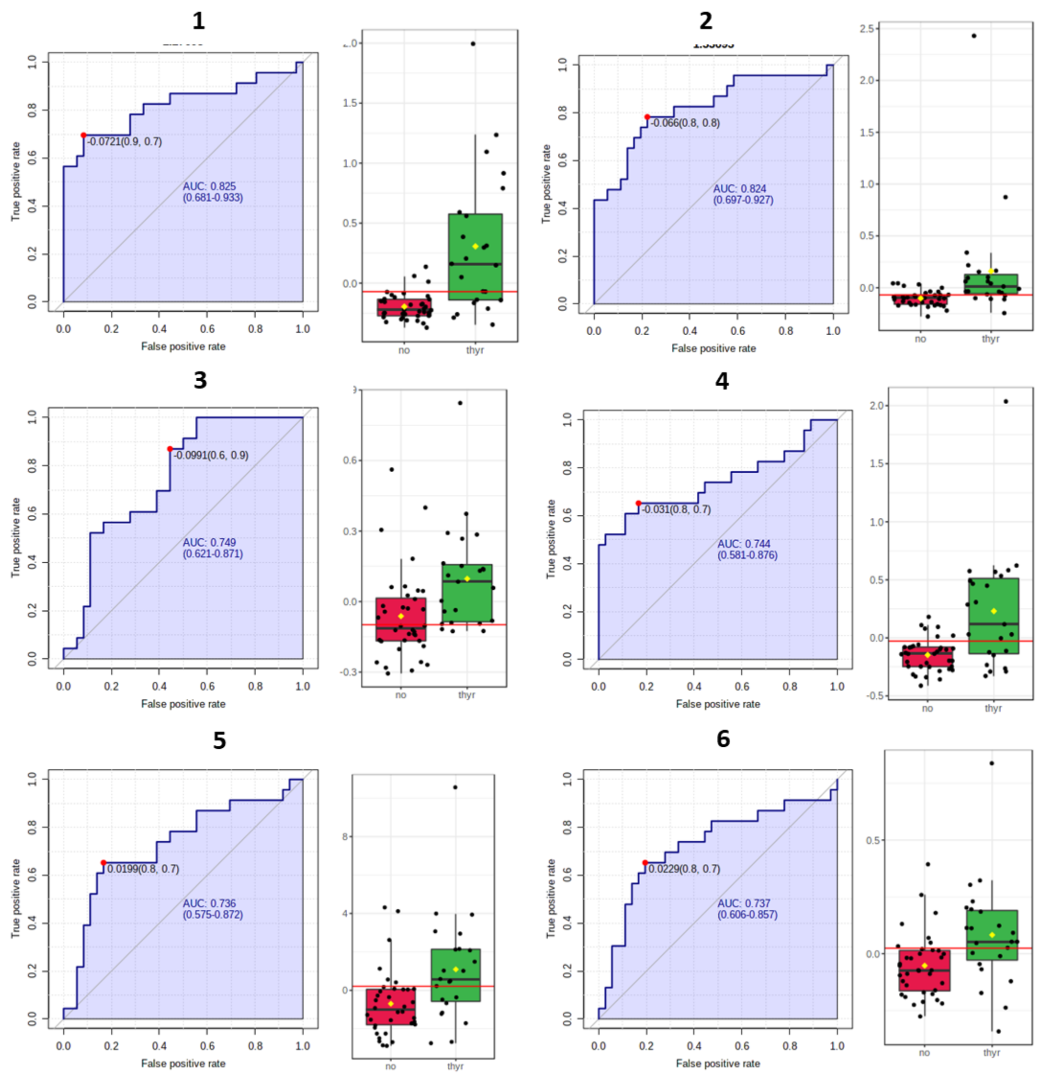

Table S1 A summary on the samples analyzed and their characteristics

class	Nationality	Parity	Maternal Age	Weight_M_Start	Weight_M_End	Height	BMI before	BMI after	Gestational Age	Birth Mode	Sex	Weight Infant	Centile	Diabetes	Hypertension	Other Pathology	Pharmaceutics	Meat (portion/ week)	Fish (portion/ week)	Dairy (portion/ week)	Legumes (portion/ week)	Eggs (portion/ week)	Smoking _before Birth (perday)	Smoking Gestation (per day)	Peculiarity	Allergy	Fruit	Vegetables	Sweets	fT4 (ng/dl)	TSH (mIU/L)
ctr	greek	3	40	54	64	1,59	21,36	25,32	294	NM	male	3350	29	no	no	glaukoma	Fe Ca folic	2	1	7	1	2	0	0	no	no	2	7	1	1,2	1,9
ctr	kazachstan	1	28	62	85	1,63	23,34	31,99	273	CS	male	3480	65	no	no	Iron deficiency anemia	Fe Ca Mg folic	2	0,5	7	2	2	0	0	no	no	7	7	2		2,1
ctr	greek	2	34	68	82	1,68	24,09	29,05	280	NM	female	3750	59	no	no	no	Fe Ca folic	3	1	7	1	0,5	0	0	no	no	7	7	1	1,1	2
ctr	albanian	1	24	75	93	1,65	27,55	34,16	269	CS	male	2630	4	no	no	no	Fe Ca folic	1	0	4	2	0	20	3	nofish, no egg	no	7	7	2	0,9	1,8
ctr	rumanian	2	32	65	80	1,7	22,49	27,68	277	NM	female	3430	52	no	no	no	Fe Ca folic	3	1	7	1	1	0	0	no	no	0	7	1	0,85	1,9
ctr	greek	2	33	96	100	1,7	33,22	34,60	280	CS	female	3740	65	no	no	medit anemia	Fe Ca folic	3	2	7	2	1	0	0	no	no	7	7	2	1,5	2,2
ctr	vietnam	1	27	47	65	1,55	19,56	27,06	259	NM	male	3460	97	no	no	no	Fe Ca	3	1	7	2	2	0	0	no	no	14	7	2	0,9	1,7
ctr	greek	2	39	48	62	1,6	18,75	24,22	265	CS	female	2570	18	no	no	no	Fe Ca	1	0,5	0	3	2	10	0	no dairy	no	3	7	2	1,2	2,3
ctr	albanian	1	27	56	65	1,57	22,72	26,37	255	CS	female	2160	10	no	no	no	Fe Ca folic	1	0	3	4	1	0	0	no fish	no	2	3	1	0,8	2,1
ctr	greek	3	25	52	58	1,6	20,31	22,66	280	NM	male	2770	4	no	no	no	Fe Ca innohep	2	0	14	1	0	0	0	no fish, no egg	no	3	7	2	0,87	2,34
ctr	russian	2	29	95	110	1,72	32,11	37,18	272	NM	female	4120	94	no	no	no	Fe Ca Mg	4	0	7	0,5	3	0	0	no fish	no	14	21	5	1,24	2,05
ctr	albanian	2	32	85	92	1,6	33,20	35,94	273	CS	male	3160	33	no	no	no	Fe Ca folic	1	1	7	2	0,5	0	0	no	no	7	14	0	0,95	2,1
ctr	greek	1	37	67	73	1,73	22,39	24,39	250	NM	male	2670	43	no	no	no	Fe salospir	2	2	7	1	4	10	3	no	no	3	7	3	1,1	1,9
ctr	syrian	1	17	57	68	1,63	21,45	25,59	280	NM	male	3220	33	no	no	no	Fe Ca folic	1	0	7	3	2	0	0	no fish	no	7	14	3	0,7	2,4
ctr	moldavian	2	29	65	80	1,7	22,49	27,68	278	NM	male	3280	25	no	no	no	Fe Ca	1	0,5	0	3	1	0	0	no dairy	no	7	14	3	0,8	1,9
ctr	rumanian	1	24	70	74	1,65	25,71	27,18	271	NM	male	2750	7	no	no	no	Fe Ca folic	3	0,5	7	0,5	3	0	0	no	no	7	7	1	1,3	2,1
ctr	greek	1	25	60	87	1,65	22,04	31,96	282	CS	male	3790	75	no	no	no	Fe Ca folic	4	1	7	0	0	10	0	no legumes	no	7	7	7	1,05	2
ctr	greek	2	41	61	63	1,6	23,83	24,61	275	NM	female	2880	15	no	no	no	Fe Ca	3	1	0	3	0	0	0	no dairy, no egg	no	3	no	7	0,95	2,1
ctr	greek	2	41	61	63	1,6	23,83	24,61	275	NM	female	2880	15	no	no	no	Fe Ca	3	1	0	3	0	0	0	no dairy, no egg	no	3	no	7	0,95	2,1
ctr	albanian	1	28	57	67	1,6	22,27	26,17	282	NM	male	3790	79	no	no	no	Fe Ca folic	2	1	7	2	1	0	0	no	no	4	14	2	0,8	2
ctr	albanian	2	27	45	65	1,5	20,00	28,89	273	CS	male	2770	17	no	no	no	Fe Ca	1	0,5	7	3	1	0	0	no	no	7	7	3	1,15	2,25
ctr	albanian	2	23	53	57	1,57	21,50	23,12	284	CS	male	3510	65	no	no	no	Fe Ca			0										0,9	2,2
ctr	greek	3	33	80	96	1,7	27,68	33,22	290	CS	male	4000	65	όχι	no	no	Fe Ca	2	1	7	1	7	0	0	no	no	7	7	7	1,25	2,4
ctr	greek	2	40	72	86	1,6	28,13	33,59	268	CS	male	3500	65	no	no	no	Fe Ca	3	1	7	1	3	2,86	0	no	no	7	7	1	0,85	2,3
ctr	albanian	2	31	55	65	1,65	20,20	23,88	275	CS	male	3550	63	no	no	no	Fe Ca	7	2	2	1	2	0	0	no	no	7	7	7	1,1	2,1
ctr	greek	1	37	59	73	1,71	20,18	24,96	273	NM	female	3420	70	no	no	multiple sclerosis	Fe Ca	7	2	7	1	2	0	0	no	no	5	7	7	1,05	2
ctr	greek	2	39	78	85	1,7	26,99	29,41	267	CS	male	3390	51	οχι	no	no	Fe Ca	4	1	7	1	7	0	0	no	no	7	7	4	1,3	1,9
ctr	greek	2	35	59	75	1,62	22,48	28,58	267	CS	female	2750	19	no	no	no	Fe Ca Mg folic	3	1	5	2	1	7	0	no	no	7	7	7	1,2	2,15
ctr	greek	1	34	56	66	1,68	19,84	23,38	272	CS	male	3600	77	no	no	no	Fe Ca	3	1	7	2	3	2	0	no	no	7	7	0	1,4	2,3

																	folic														
ctr	greek	1	34	65	80	1,6	25,39	31,25	281	CS	male	3620	62	οχι	no	no	Fe Ca folic	6	1	7	1	2	0	0	no	no	7	7	0,75	1,3	1,8
ctr	albanian	2	30	45	60	1,54	18,97	25,30	277	AE	female	3030	45	no	no	no	Fe Ca	1	2	3	2	2	0	0	no	paraketamoli	5	7	1	0,9	2,2
ctr	greek	1	48	76	82	1,62	28,96	31,25	275	CS	male	3370	42	όχι	no	no	Fe Ca	3	0	3	1	0	0	0	no fish, no egg	egg	1	2	4	1,2	2,1
ctr	albanian	3	35	85	102	1,74	28,08	33,69	268	NM	female	3150	33	οχι	no	no	Fe Ca folic	2	0,25	7	2	1	0	0	no	no	7	1	1	1,1	2,15
ctr	albanian	1	26	47	62	1,54	19,82	26,14	277	AE	male	3230	56	no	no	no	Fe Ca folic	2	0	7	1	3	5	0	no	no	7	7	1	0,85	2,3
ctr	albanian	2	27	51	67	1,61	19,68	25,85	282	NM	female	4060	95	no	no	no	Fe Ca Mg folic	2	2	7	2	7	0	0	no	no	7	7	7	1,4	2,2
ctr	greek	8	40	70	82	1,59	27,69	32,44	267	NM	male	2700	6	no	no	no	Fe Ca Mg innohep	2	1	7	2	1	0	0	no	nafthaline, levanta	7	5	4	0,95	2,1
ctr	moldavian	2	43	83	100	1,69	29,06	35,01	273	NM	female	3950	91	no	no	no	Fe Ca Mg folic	7	0,5	7	0,5	3	0	0	no	no	7	7	7	1,3	2,05
ctr	moldavian	2	34	64	80	1,64	23,80	29,74	278	NM	male	3360	34	no	no	no	Fe Ca Mg	5	7	3										1,1	2,3
ctr	greek	4	40	70	75	1,65	25,71	27,55	270	CS	male	3720	73	no	no	no	Fe Ca Mg	5	0,25	7	2	1	1	0	milk coconut	no	2	7	7	1,2	2,25
ctr	greek	2	38	55	81	1,74	18,17	26,75	271	CS	male	3900	87	no	no	no	no	4	2	7	1	2	0	0	no	no	7	14	5	1,1	2,1
ctr	rumanian	1	25	53	73	1,7	18,34	25,26	263	CS	female	2960	60	no	no	no	no	1	2	7	2	0	0	0	no egg	strawberry, mandarines	7	7	0	1,15	2,1
ctr	greek	4	35	68	77	1,6	26,56	30,08	270	NM	male	3320	44	GDM (insul)	no	no	Fe insulin	3	1	7	3	4	3	0	no	no	7	7	0	1,1	1,4
ctr	greek	1	29	74	79	1,65	27,18	29,02	274	NM	female	3280	50	no	no	no	no	3	0	2	2	1	0	0	no fish	no	14	7	7	0,9	2,3
ctr	albanian	2	33	62	77	1,56	25,48	31,64	263	NM	female	3990	99	no	no	no	Fe Ca folic	2	1	14	1	3	0	0	no	no	3	14	3	0,85	2,1
ctr	albanian	1	20	47	60	1,7	16,26	20,76	287	NM	male	3220	33	no	no	no	Fe Ca	4	0,25	7	2	0	0	0	no egg	no	7	14	2	0,9	2,15
ctr	greek	2	35	94	112	1,68	33,30	39,68	280	NM	female	2910	6	no	no	no	Fe Ca Mg folic	2	1	7	1	2	0	0	no	no	7	7	7	1	2,35
ctr	rumanian	5	27	72	84	1,7	24,91	29,07	277	NM	female	4060	92	no	no	no	Fe Ca folic	2	1	7	1	2	0	0	no	no	7	4	7	1,1	2,1
ctr	greek	2	36	59	72	1,67	21,16	25,82	265	NM	male	2940	20	no	no	no	Fe Ca Mg folic	7	2	7	3	4	0	0	no	cat	3	7	0	1,1	2,1
ctr	greek	1	27	110	112	1,7	38,06	38,75	268	NM	male	2830	10	no	no	no	Fe Ca Mg folic Aspirin	3	1	14	3	1	0	0	no	no	4	7	3	1,05	2,1
ctr	albanian	2	29	56	80	1,6	21,88	31,25	283	NM	female	3400	54	no	no	no	Fe Ca folic	2	1	7	2	2	0	0	no	no	3	3	2	0,9	2,3
thyr	greek	1	44	61	69	1,7	21,11	23,88	273	CS	male	2630	2	no	no	hypothyr	Fe Ca Mg t4	2	0,5	7	4	0	0	0	no	no	28	7	0	0,6	4,3
thyr	greek	1	29	72	84	1,65	26,45	30,85	256	NM	female	3090	79	no	no	hypothyr	Fe Ca Thyrhormone	2	1	7	2	2	0	0	no	no	7	7	3	1,4	2,2
thyr	greek	1	29	61	64	1,67	21,87	22,95	276	NM	male	2960	14	no	no	hyperthyr	Fe Ca prothouril	3	0,25	7	1	1	0	0	no	no	1	7	1	0,7	2,2
thyr	greek	4	27	53	68	1,58	21,23	27,24	266	CS	female	3150	66	no	no	hypothyr	Fe Ca Mg t4	4	0	7	0	2	0	0	no fish, no legumes	no	7	7	1	0,9	2,7
thyr	greek	1	33	85	100	1,7	29,41	34,60	272	CS	femal	2820	12	no	no	hypothyr	Fe Ca	1	2	7	1	0	1	2,5	no	no	7	7	0	1,3	2

											e						Mg t4														
thyr	greek	1	27	57	70	1,6	22,27	27,34	282	CS	male	3580	64	no	no	hypothyr, asthma	Fe Ca folic	3	1	7	3	3	0	0	every day swee ts	no	7	14	7	1,3	2,4
thyr	greek	2	33	57	68	1,63	21,45	25,59	253	CS	femal e	3740	99	GDM (diet)	no	hypothyr	Fe CaMg folic t4	3	0,5	7	0	4	5	3	no legu mes, no veget ables	no	21	no	0	0,5	2,9
thyr	albanian	1	28	52	60	1,58	20,83	24,03	275	CS	femal e	3390	74	no	no	hypothyr	Fe Ca t4	1	2	7	0,25	2	12	0	no	akare a	7	7	5	0,9	3,2
thyr	greek	1	30	73	87	1,73	24,39	29,07	285	CS	male	4250	85	no	no	hypothyr	Fe Ca t4	2	2	7	2	3	0	0	no	no	7	7	0	1,1	2,5
thyr	albanian	1	23	56	76	1,52	24,24	32,89	277	AE	male	3720	83	GDM (insu l)	no	hypothyr	Fe Ca T4 62 insulin	4	0,5	7	1	3	0	0	no	no	7	7	7	0,9	2,6
thyr	greek	2	38	67	78	1,7	23,18	26,99	264	AE	male	3980	94	no	no	hypothyr	Fe Ca t4 50μg	3	2	1	1	2	0	0	no	no	7	5	3	0,72	3,6
thyr	greek	1	24	80	92	1,55	33,30	38,29	270	CS	femal e	3070	65	no	no	hypothyr	Fe Ca folic t4	4	2	5	1	1	15	4	no	no	7	2	4	1,1	2,3
thyr	rumania n	5	38	76	82	1,6	29,69	32,03	245	CS	femal e	3460	98	no	no	hypothyr	Fe Ca Mg folic t4 75	3	1	14	1	2	0	0	no	no	7	7	3	0,6	3,6
thyr	rumania n	4	32	60	90	1,63	22,58	33,87	270	NM	femal e	3360	65	no	no	hypothyr	Fe Ca T4	2	1	7	1	1	20	4	no	no	7	7	1	0,6	3,2
thyr	albanian	1	27	54	70	1,69	18,91	24,51	271	NM	femal e	3040	50	no	no	hashimotto	Fe CaMg folict4	3	1	7	1	2	12	0,43	no	no	7	7	0	0,8	2,7
thyr	greek	1	39	64	84	1,64	23,80	31,23	279	CS	male	3940	83	GDM (diet)	no	hypothyr	Fe Ca folic t4	3	1	7	2	1	0	0	no	no	14	7	2	0,92	2,1
thyr	filippine s	1	32	65	79	1,67	23,31	28,33	286	NM	femal e	2940	17	GDM (diet)	no	hypothyr	Fe Ca	4	2	14	2	3	0	0	no	no	7	14	3	1	2,6
thyr	filippine s	2	34	63	75	1,52	27,27	32,46	269	NM	male	3410	62	no	no	hashimotto	Fe Ca folic	1	2	7	0,5	3	0	0	no	no	7	7	0,5	0,54	2,9
thyr	greek	1	26	57	77	1,6	22,27	30,08	274	NM	femal e	3590	86	no	no	hypothyr	Fe Ca folic t4 75	2	1	7	2	3	0	0	no	no	7	7	2	0,7	3,1
thyr	greek	2	37	100	115	1,7	34,60	39,79	272	CS	male	4600	97	no	no	hypothyr	Fe Ca folic t4	2	0	7	2	4	0	0	no fish , often fast food	no	14	5	7	1,2	4,6
thyr	greek	1	31	62	78	1,75	20,24	25,47	249	CS	male	2890	79	no	no	hypothyr	Fe Ca t4	3	1	7	1	1	5	0	no	no	14	7	4	0,8	2,96
thyr	filippine s	1	30	47	67	1,58	18,83	26,84	273	CS	male	3070	44	no	no	hypothyr	Fe Ca	1	2	7	2	1	0	0	usual ly sushi	no	14	7	2	0,9	2,9
thyr	greek	3	32	70	78	1,56	28,76	32,05	272	CS	male	3400	43	GDM (insu l)	no	hypothyr	Fe Ca T4 62	3	1	7	3	2	0	0	no	no	no	7	0	0,5	2,8
thyr	albanian	3	29	65	77	1,62	24,77	29,34	251	NM	male	3860	99	no	no	hypothyr	Fe Ca folic t4	3	1	7	1	2	0	0	no	no	7	7	1	0,9	4
thyr	greek	1	37	58	69	1,63	21,83	25,97	272	AE	femal e	3150	52	no	no	hypothyr , glaukoma	Fe Ca Mg t4	2	1	7	1	2	0	0	no	no	3	7	3		2,9
thyr	georgia n	1	31	53	75	1,59	20,96	29,67	281	NM	femal e	3450	71	no	no	hypothyr	Fe Ca t4	1	0	7	3	3	20	10	no fish	no	7	7	0	0,8	2,6
thyr	greek	4	39	120	134	1,61	46,29	51,70	273	CS	male	3660	56	GDM (insu l)	no	thyr	Fe folic t4 insulin	2	1	7	1	2	0	0	no	peicil in	1	7	2	0,9	3,21
thyr	albanian	1	36	54	70	1,59	21,36	27,69	263	NM	male	3070	62	no	no	thyr	t4 75	2	1	7	1	3	0	0	no	bee	7	7	7	0,9	2,3
thyr	ukrania n	1	30	58	80	1,69	20,31	28,01	267	CS	femal e	3340	78	no	no	hypothyr	Fe Ca folic t4	4	0,25	7	0,75	4	10	2	no	no	7	4	7	0,6	4,4
thyr	greek	1	23	70	83	1,7	24,22	28,72	279	CS	femal e	3170	31	no	no	hypothyr	Fe Ca Mg t4	2	0	7	2	3	0,43	0,43	no fish	pepp er	4	3	2	0,9	2,1
thyr	filippine s	1	32	60	75	1,55	24,97	31,22	280	CS	femal e	3070	33	no	no	hyperthyr	Fe Ca methima zole	1	2	7	1	1	0	0	no	no	7	7	0	0,7	2,6
thyr	albanian	2	25	79	91	1,63	29,73	34,25	274	CS	male	3450	44	no	no	hypothyr	Fe Ca	4	1	7	0	3	0	0	no	no	5	7	4	0,5	2,6

																	folic t4									legu mes							
thyr	greek	1	37	53	67	1,58	21,23	26,84	276	CS	femal e	3170	54	GDM (insu l)	no	hypothyr	Fe Ca folict4 insulin	2	1	7	1	0	10	0	no egg	no	7	7	1	0,53	3,1
thyr	greek	1	42	62	78	1,63	23,34	29,36	271	CS	femal e	3180	54	GDM (insu l)	no	hypothyr	t4 125 salospir innohepi nsulin	3	1	7	2	3	0	0	no	no	7	7	0	1,04	2,9
thyr	greek	1	32	58	78	1,75	18,94	25,47	285	NM	femal e	3320	39	no	no	hypothyr	Fe Ca t4 62	3	1	7	2	2	10	0	no	no	14	7	1	0,9	2,6
thyr	greek	2	36	54	59	1,62	20,58	22,48	259	CS	femal e	2770	48	no	no	hypothyr	Fe Ca t4 137	2	1	7	3	3	5	0	no	no	4	7	4	0,95	2,77
thyr	greek	2	45	70	85	1,59	27,69	33,62	277	CS	femal e	3370	48	no	no	hypothyr	Fe Ca folict4 125	1	2	4	1	2	0	0	no	no	7	14	0	1,2	3,8
thyr	greek	1	24	60	76	1,65	22,04	27,92	266	CS	male	2430	2	no	no	hypothyr	Fe Ca Mg t4 62	3	0,25	7	4	0	20	0	no egg	no	4	0,5	3	0,8	2,5
thyr	greek	1	35	54	71	1,6	21,09	27,73	272	CS	femal e	2740	19	no	no	hyperthyr	Fe Ca folic unimazo le	2	0	7	1	7	7	0	no fish	no	7	2	4	0,7	3

Table S2 Summary of metabolites in both biofluids with a p-value < 0.005

Metabolite IUPAC (serum)	Metabolite (serum)	V	p.value	-LOG10(p)	FDR
propane-1,2,3-triol	glycerol	891	1,22E-10	9,9122	8,08E-08
unsaturated lipid	unsaturated lipid	91	1,96E-09	8,7075	1,98E-07
(3β)-cholest-5-en-3-ol-VLDL	cholesterol-VLDL	134	2,06E-07	6,686	2,11E-06
2-aminopropanoic acid	alanine	664	0,009303	2,0314	0,029354
methanol	methanol	304	0,012492	1,9034	0,038257
LDL2/VLDL2	LDL2/VLDL2	108	1,42E-08	7,8473	2,84E-07
2-amino-3-(4-hydroxyphenyl)propanoic acid	tyrosine	805	4,83E-02	1,3162	8,99E-01
1-methylhistidine	1-methylhistidine	718	8,05E-03	2,0943	8,99E-01
2-amino-4-(methylsulfanyl)butanoic acid	methionine	793	3,86E-02	1,413	8,99E-01
2-amino-3-phenylpropanoic acid	L-phenylalanine	750	1,63E-02	1,7873	8,99E-01
**Metabolite IUPAC (milk)**	**Metabolite (milk)**	**V**	**p.value**	**-LOG10(p)**	**FDR**
Fucosyl moieties	Fucosyl moieties	129	2,91E-06	5,5364	0,00235
2-amino-3-hydroxybutanoic acid	threonine	148	1,55E-05	4,8084	0,00235
2-hydroxypropanoic acid	lactate	212	0,00139	2,857	0,037353
[2-(trimethylazaniumyl)ethoxy]phosphonic acid	0-phosphocholine	205	0,000916	3,0383	0,027568
(2-hydroxyethyl)trimethylazanium	choline	227	0,003224	2,4916	0,066046
(2-{[(2R)-2,3-dihydroxypropyl phosphono]oxy}ethyl)trimethylazanium	glycerophospocholine	224	0,002894	2,5385	0,059037

Table S3 Result from Pathway Analysis on the Serum samples

Pathway Name	Match Status	p	-log(p)	Holm p	FDR	Impact
Phenylalanine, tyrosine and tryptophan biosynthesis	2/ 4	1,045E-04	39.809	0.0087786	0.0087786	1.0
Phenylalanine metabolism	2/ 10	7,737E-04	31.114	0.064219	0.025056	0.35714
Aminoacyl-tRNA biosynthesis	3/ 48	8,949E-04	30.483	0.073378	0.025056	0.0
Ubiquinone and other terpenoid-quinone biosynthesis	1/9	0.04002	13.977	1.0	0.84042	0.0
Histidine metabolism	1/ 16	0.07019	11.537	1.0	0.98266	0.0
Glycerolipid metabolism	1/ 16	0.07019	11.537	1.0	0.98266	0.23676
Pyruvate metabolism	1/ 22	0.0954	10.205	1.0	1.0	0.06065
Glycolysis / Gluconeogenesis	1/ 26	0.11188	0.95125	1.0	1.0	0.02906
Galactose metabolism	1/ 27	0.11596	0.9357	1.0	1.0	0.0
Glyoxylate and dicarboxylate metabolism	1/ 32	0.13611	0.8661	1.0	1.0	0.0
Cysteine and methionine metabolism	1/ 33	0.1401	0.85357	1.0	1.0	0.10446
Tyrosine metabolism	1/ 42	0.17525	0.75635	1.0	1.0	0.13972

Table S4 Result from Pathway Analysis on the colostrum samples

Pathway Name	Match Status	p	-log(p)	Holm p	FDR	Impact
Glycerophospholipid metabolism	3/ 36	1,12E-04	39.523	0.009375	0.009375	0.08333
Glycine, serine and threonine metabolism	2/ 33	0.0042247	23.742	0.35065	0.17744	0.0
Valine, leucine and isoleucine biosynthesis	1/ 8	0.025574	15.922	1.0	0.71608	0.0
Ether lipid metabolism	1/ 20	0.062952	1.201	1.0	1.0	0.0
Pyruvate metabolism	1/ 22	0.069068	11.607	1.0	1.0	0.0
Glycolysis / Gluconeogenesis	1/ 26	0.081205	10.904	1.0	1.0	0.0
Aminoacyl-tRNA biosynthesis	1/ 48	0.14572	0.83649	1.0	1.0	0.0

Table S5 Resonance peak identification of 37 metabolites with the Metaboneer software in serum with report of their fold changes

Metabolite	Metabolite (IUPAC)	Ser1	Ser2	Ser3	Ser4	Ser5	Ser6	Ser7	Ser8	Ser9	Ser10	Ser11	Ser12	Ser13	Ser14	Ser15	Ser16	Ser17	Ser18	Ser19	Ser20	Ser21	Ser22	Ser23	Ser24	Ser25	Ser26	Ser27	Ser28	Ser29	Ser30	Ser31	Ser32	Ser33	Ser34	Ser35	Ser36	Ser37	Ser38	Ser39	Ser40	Ser41	Ser42	Ser43	Ser44
**Citric acid**	**2-hydroxypropane-1,2,3-tricarboxylic acid**	0,059	0,000	0,000	0,091	0,000	0,000	0,075	0,000	0,000	0,077	0,000	0,000	0,000	0,000	0,050	0,000	0,000	0,000	0,000	0,000	0,000	0,000	0,000	0,000	0,000	0,000	0,000	0,000	0,000	0,000	0,000	0,000	0,000	0,000	0,000	0,000	0,000	0,000	0,000	0,000	0,000	0,000	0,000	0,000
**L-Lactic acid**	**(2S)-2-hydroxypropanoic acid**	2,402	1,188	3,180	1,825	2,620	1,890	3,010	2,350	6,272	2,638	2,091	2,045	4,249	2,219	2,182	1,936	3,251	2,650	3,817	2,247	0,497	2,129	1,871	1,679	1,999	1,309	0,506	1,584	2,138	0,589	0,657	2,214	0,555	0,535	0,570	0,896	0,928	1,118	0,584	0,428	1,635	1,787	0,578	0,566
**L-Alanine**	**(2S)-2-aminopropanoic acid**	2,354	0,667	2,621	2,843	2,524	2,062	2,900	0,653	3,598	3,056	0,511	2,905	4,156	1,852	2,206	3,001	2,867	2,585	2,998	1,883	0,000	1,948	1,484	1,694	1,672	1,112	0,000	0,518	1,454	0,000	0,000	1,655	0,000	0,000	0,000	1,134	1,175	1,383	0,000	0,000	1,572	1,723	0,000	0,000
**L-Valine**	**(2S)-2-amino-3-methylbutanoic acid**	1,275	1,505	2,175	1,133	1,663	2,103	1,349	1,599	1,814	2,314	1,626	1,977	2,388	1,044	1,266	2,060	1,890	1,717	1,661	1,557	1,122	1,270	1,400	1,233	1,361	0,922	1,078	0,748	1,008	1,216	1,106	0,795	0,904	1,061	1,303	0,929	1,038	1,071	1,114	0,973	1,356	1,435	1,186	1,440
**D-Glucose**	**(2R,3R,4S,5S,6R)-6-(hydroxymethyl)oxane-2,3,4,5-tetrol**	14,247	12,640	19,421	17,936	14,832	15,205	23,382	18,328	15,186	18,582	19,758	16,400	25,876	10,190	11,628	11,466	14,179	15,939	18,246	11,194	6,621	9,317	10,645	10,294	9,736	7,554	7,294	7,193	7,193	8,393	5,240	8,578	5,814	7,289	7,467	7,711	5,534	6,235	4,995	5,101	6,577	9,278	5,340	8,114
**L-Leucine**	**(2S)-2-amino-4-methylpentanoic acid**	0,702	0,653	0,797	0,634	0,813	0,977	0,580	0,938	1,130	0,792	1,004	1,091	1,210	0,541	0,635	0,908	0,908	1,026	0,889	1,159	1,033	0,401	1,212	0,668	0,650	0,481	0,314	0,520	0,918	0,651	0,344	0,943	0,647	0,808	0,711	0,918	0,610	0,319	0,840	0,596	0,677	1,118	0,562	0,909
**L-Isoleucine**	**(2S,3S)-2-amino-3-methylpentanoic acid**	0,000	0,000	0,000	0,000	0,000	0,000	0,000	0,000	0,780	0,813	0,910	0,000	0,000	0,492	0,347	0,724	0,000	0,000	0,000	0,639	0,464	0,000	0,000	0,511	0,000	0,620	0,333	0,219	0,765	0,000	0,000	0,281	0,000	0,000	0,517	0,534	0,000	0,615	0,000	0,000	0,000	0,000	0,000	0,383
**L-Phenylalanine**	**(2S)-2-amino-3-phenylpropanoic acid**	0,025	0,043	0,027	0,027	0,019	0,025	0,025	0,025	0,166	0,019	0,036	0,047	0,048	0,014	0,020	0,013	0,037	0,007	0,033	0,122	0,000	0,000	0,089	0,463	0,374	0,205	0,000	0,541	0,000	0,000	0,280	0,315	0,000	0,000	0,000	0,283	0,240	0,243	0,244	0,304	0,271	0,329	0,000	0,000
**Formic acid**	**formic acid**	0,009	0,013	0,008	0,013	0,005	0,012	0,014	0,005	0,010	0,013	0,010	0,010	0,012	0,016	0,015	0,011	0,013	0,009	0,005	0,032	0,060	0,042	0,048	0,038	0,061	0,044	0,030	0,042	0,059	0,041	0,057	0,051	0,048	0,047	0,074	0,050	0,027	0,045	0,049	0,035	0,028	0,027	0,069	0,035
**L-Histidine**	**(2S)-2-amino-3-(1H-imidazol-5-yl)propanoic acid**	0,054	0,064	0,081	0,046	0,061	0,057	0,057	0,056	0,104	0,067	0,052	0,056	0,093	0,012	0,038	0,001	0,078	0,060	0,055	0,105	0,064	0,059	0,070	0,110	0,115	0,103	0,055	0,063	0,148	0,053	0,099	0,063	0,068	0,097	0,138	0,069	0,101	0,101	0,067	0,094	0,246	0,062	0,111	0,128
**L-Tyrosine**	**(2S)-2-amino-3-(4-hydroxyphenyl)propanoic acid**	0,073	0,038	0,099	0,051	0,070	0,063	0,082	0,074	0,058	0,167	0,067	0,082	0,076	0,055	0,063	0,121	0,083	0,052	0,084	0,245	0,000	0,253	0,267	0,262	0,286	0,000	0,000	0,233	0,299	0,256	0,247	0,228	0,000	0,000	0,293	0,255	0,000	0,000	0,236	0,000	0,271	0,257	0,239	0,282
**Choline**	**(2-hydroxyethyl)trimethylazanium**	0,125	0,021	0,000	0,128	0,129	0,271	0,121	0,017	1,081	0,462	0,465	0,229	1,125	0,071	0,115	0,212	0,201	0,159	0,019	0,645	0,178	0,000	1,321	0,302	0,427	0,293	0,000	0,149	0,163	0,000	0,333	0,472	0,134	0,238	0,171	0,452	0,432	0,450	0,265	0,212	0,276	0,394	0,205	0,000
**Phosphorylcholine**	**[2-(trimethylazaniumyl)ethoxy]phosphonic acid**	0,039	0,052	0,061	0,034	0,039	0,093	0,054	0,032	0,067	0,085	0,088	0,034	0,073	0,042	0,046	0,059	0,038	0,047	0,075	0,065	0,000	0,099	0,000	0,046	0,000	0,073	0,078	0,047	0,000	0,081	0,000	0,112	0,038	0,073	0,000	0,078	0,071	0,067	0,059	0,074	0,072	0,078	0,073	0,098
**Acetone**	**propan-2-one**	0,096	0,025	0,089	0,034	0,047	0,146	0,051	0,044	0,113	0,163	0,402	0,117	0,088	0,063	0,081	0,120	0,083	0,189	0,035	0,175	0,104	0,126	0,221	0,110	0,132	0,087	0,126	0,086	0,128	0,104	0,112	0,098	0,091	0,088	0,138	0,177	0,223	0,158	0,087	0,258	0,101	0,150	0,124	0,166
**Acetic acid**	**acetic acid**	0,051	0,066	0,053	0,063	0,049	0,089	0,067	0,083	0,100	0,055	0,117	0,088	0,127	0,072	0,069	0,140	0,081	0,109	0,094	0,088	0,073	0,154	0,277	0,157	0,098	0,062	0,070	0,173	0,094	0,160	0,094	0,085	0,080	0,063	0,096	0,092	0,090	0,183	0,059	0,073	0,062	0,294	0,065	0,107
**Acetoacetic acid**	**3-oxobutanoic acid**	0,049	0,050	0,103	0,060	0,045	0,061	0,095	0,054	0,268	0,083	0,049	0,070	0,088	0,033	0,051	0,052	0,064	0,078	0,143	0,100	0,088	0,095	0,057	0,088	0,082	0,057	0,078	0,061	0,079	0,111	0,052	0,085	0,059	0,106	0,075	0,058	0,057	0,062	0,056	0,199	0,087	0,057	0,075	0,064
**Methylamine**	**methanamine**	0,009	0,008	0,001	0,027	0,003	0,008	0,010	0,006	0,022	0,024	0,003	0,002	0,000	0,045	0,015	0,003	0,009	0,021	0,002	0,057	0,053	0,061	0,075	0,056	0,068	0,060	0,058	0,050	0,085	0,056	0,059	0,079	0,036	0,052	0,082	0,072	0,052	0,060	0,061	0,059	0,052	0,060	0,059	0,063
**Dimethylamine**	**dimethylamine**	0,014	0,011	0,019	0,018	0,005	0,016	0,015	0,005	0,008	0,018	0,004	0,014	0,003	0,012	0,005	0,021	0,013	0,031	0,016	0,073	0,10	0,12	0,000	0,051	0,00	0,04	0,00	0,07	0,05	0,05	0,03	0,11	0,05	0,00	0,00	0,08	0,05	0,03	0,05	0,00	0,05	0,118	0,04	0,07

																						4	6			0	5	0	2	5	4	9	7	2	0	0	2	8	5	0	0	6		3	1
**Creatine**	**2-(N-methylcarbamimidamido)acetic acid**	0,006	0,016	0,013	0,013	0,015	0,097	0,024	0,074	0,024	0,092	0,005	0,007	0,002	0,000	0,072	0,078	0,006	0,031	0,010	0,089	0,000	0,000	0,000	0,000	0,000	0,061	0,058	0,046	0,061	0,073	0,000	0,048	0,000	0,000	0,000	0,055	0,000	0,000	0,000	0,000	0,045	0,000	0,000	0,000
**Creatinine**	**2-imino-1-methylimidazolidin-4-one**	0,230	0,350	0,265	0,271	0,273	0,183	0,385	0,130	0,232	0,149	0,326	0,411	0,348	0,221	0,166	0,197	0,332	0,250	0,231	0,249	0,200	0,173	0,705	0,193	0,201	0,104	0,082	0,121	0,158	0,086	0,153	0,149	0,138	0,189	0,216	0,124	0,119	0,208	0,168	0,195	0,153	0,000	0,121	0,240
**Glycine**	**2-aminoacetic acid**	0,564	0,397	0,282	0,481	0,489	0,423	0,513	0,313	0,761	0,406	0,348	0,624	0,789	0,423	0,344	0,446	0,514	0,445	0,391	0,292	0,187	0,278	0,213	0,243	0,313	0,249	0,388	0,245	0,168	0,506	0,293	0,347	0,222	0,439	0,206	0,330	0,316	0,333	0,275	0,226	0,256	0,351	0,218	0,251
**Methanol**	**methanol**	0,289	0,354	0,486	0,000	0,271	0,000	1,392	0,362	0,432	0,263	0,000	0,000	0,000	0,393	0,155	0,258	0,000	0,000	0,317	0,000	0,000	0,335	0,000	0,000	0,000	0,000	0,000	0,000	0,000	0,000	0,000	0,000	0,000	0,000	0,000	0,000	0,000	0,000	0,000	0,000	0,000	0,000	0,000	0,447
**Betaine**	**(carboxymethyl)trimethylazanium**	0,018	0,137	0,121	0,049	0,030	0,060	0,050	0,032	0,033	0,065	0,062	0,052	0,164	0,029	0,074	0,093	0,034	0,033	0,094	0,075	0,065	0,109	0,071	0,077	0,058	0,068	0,082	0,038	0,059	0,084	0,044	0,082	0,064	0,088	0,057	0,082	0,064	0,075	0,057	0,082	0,059	0,075	0,064	0,095
**3-Hydroxybutyric acid**	**(3R)-3-hydroxybutanoic acid**	0,006	0,087	0,011	0,048	0,076	0,552	0,004	0,010	0,222	0,548	1,306	0,010	0,559	0,003	0,509	0,377	1,051	0,670	0,005	0,000	3,159	0,000	0,000	0,000	0,000	0,000	1,333	0,000	0,000	2,124	1,075	0,000	1,147	1,093	1,716	0,177	0,637	0,097	1,017	1,166	0,000	0,000	1,432	1,117
**2-Hydroxybutyric acid**	**(2S)-2-hydroxybutanoic acid**	0,000	0,000	0,000	0,000	0,000	0,000	0,000	0,000	0,000	0,000	0,000	0,000	0,000	0,000	0,000	0,000	0,000	0,000	0,000	0,000	0,000	0,000	0,000	0,000	0,000	0,000	0,000	0,000	0,000	0,000	0,000	0,000	0,000	0,000	0,000	0,000	0,000	0,000	0,000	0,000	0,000	0,000	0,000	0,000
**Isobutyric acid**	**2-methylpropanoic acid**	0,177	0,027	0,104	0,007	0,111	0,083	0,011	0,093	0,067	0,084	0,007	0,110	0,032	0,195	0,005	0,082	0,004	0,142	0,856	0,222	0,000	0,000	0,000	0,000	0,000	0,193	0,000	0,000	0,000	0,000	0,179	0,000	0,294	0,659	0,555	0,771	0,944	0,288	0,099	0,410	0,000	0,000	0,274	0,367
**(S)-3-Hydroxyisobutyric acid**	**(2S)-3-hydroxy-2-methylpropanoic acid**	0,077	0,075	0,109	0,100	0,004	0,027	0,124	0,005	0,261	0,014	0,106	0,003	0,117	0,109	0,005	0,012	0,005	0,014	0,073	0,000	0,000	0,000	0,000	0,000	0,000	0,000	0,000	0,000	0,000	0,000	0,411	0,000	0,000	0,000	0,000	0,000	0,000	0,000	0,000	0,000	0,000	0,000	0,000	0,250
**L-Glutamine**	**(2S)-2-amino-4-carbamoylbutanoic acid**	0,000	0,636	0,628	0,520	0,658	0,722	0,637	0,039	0,071	0,833	0,592	0,469	0,671	0,571	1,142	0,450	0,549	0,817	0,554	1,506	0,741	1,145	0,391	1,210	1,041	0,299	0,947	0,280	0,650	0,382	0,304	1,215	1,228	0,328	1,140	0,336	0,976	0,935	0,876	0,509	1,072	0,898	1,150	0,375
**L-Glutamic acid**	**(2S)-2-aminopentanedioic acid**	0,714	0,286	1,044	0,214	0,657	0,658	0,000	0,783	1,212	0,000	0,668	0,298	0,843	0,401	0,000	0,934	0,000	0,000	0,000	1,230	0,000	0,000	0,000	0,000	0,777	0,000	0,000	0,000	0,803	0,000	0,000	0,000	0,000	0,000	0,905	0,000	0,000	0,000	0,000	0,000	0,000	0,000	0,000	0,000
**L-Aspartic acid**	**(2S)-2-aminobutanedioic acid**	0,067	0,032	0,134	0,095	0,088	0,152	0,113	0,081	0,108	0,100	0,101	0,134	0,039	0,121	0,074	0,057	0,114	0,193	0,053	0,000	0,000	0,000	0,000	0,000	0,000	0,000	0,000	0,000	0,000	0,000	0,000	0,000	0,000	0,000	0,000	0,000	0,000	0,000	0,000	0,000	0,000	0,000	0,000	0,000
**L-Asparagine**	**(2S)-2-amino-3-carbamoylpropanoic acid**	0,028	0,033	0,053	0,056	0,044	0,138	0,112	0,031	0,051	0,079	0,030	0,082	0,017	0,050	0,032	0,145	0,051	0,115	0,037	0,000	0,000	0,000	0,000	0,000	0,000	0,000	0,000	0,000	0,000	0,000	0,000	0,000	0,000	0,000	0,000	0,255	0,000	0,160	0,415	0,000	0,000	0,000	0,184	0,000
**L-Threonine**	**(2S,3R)-2-amino-3-hydroxybutanoic acid**	0,175	0,137	0,633	0,324	0,150	0,583	0,184	0,005	0,486	0,164	0,128	0,162	0,199	0,113	0,371	0,004	0,182	0,508	0,124	0,462	0,339	0,422	0,314	0,483	0,168	0,270	0,154	0,373	0,354	0,482	0,371	0,172	0,148	0,464	0,513	0,512	0,274	0,288	0,455	0,484	0,397	0,583	0,471	0,802
**D-Lysine**	**(2R)-2,6-diaminohexanoic acid**	0,295	0,218	0,492	0,064	0,137	0,000	0,174	0,162	0,270	0,000	0,153	0,314	0,208	0,138	0,149	0,376	0,000	0,173	0,152	0,301	0,000	0,000	0,317	0,291	0,000	0,000	0,000	0,253	0,000	0,000	0,000	0,000	0,000	0,000	0,000	0,000	0,000	0,260	0,000	0,000	0,306	1,078	0,000	0,000
**L-Arginine**	**(2S)-2-amino-5-carbamimidamidopentanoic acid**	0,358	0,246	0,131	0,251	0,962	0,000	0,090	0,081	2,196	0,000	0,098	0,074	5,358	2,184	0,060	0,274	0,000	0,413	0,267	0,821	0,000	0,000	1,420	2,075	0,000	0,000	0,000	0,454	0,000	0,000	0,000	0,000	0,000	0,000	0,000	0,000	0,000	0,572	0,000	0,000	1,341	2,589	0,000	0,000
**L-Methionine**	**(2S)-2-amino-4-(methylsulfanyl)butanoic acid**	0,370	0,225	0,024	0,027	0,440	0,055	0,035	0,409	0,021	0,046	0,025	0,386	0,044	0,267	0,612	0,037	0,280	0,044	0,030	0,000	0,000	0,000	0,000	0,000	0,000	0,000	0,000	0,000	0,000	0,000	0,000	0,000	0,000	0,000	0,000	0,000	0,000	0,000	0,000	0,000	0,000	0,204	0,000	0,000
**Syringic acid**	**4-hydroxy-3,5-dimethoxybenzoic acid**	0,000	1,161	0,000	0,000	1,450	0,000	2,100	0,000	0,000	0,000	0,000	0,000	0,000	0,828	0,000	0,000	0,000	1,493	0,000	0,000	0,000	0,000	0,000	0,000	0,000	0,000	0,000	0,000	0,000	0,000	0,000	0,000	0,000	0,000	0,000	0,000	0,000	0,000	0,000	0,000	0,000	0,000	0,000	0,000
**Pyruvic acid**	**2-oxopropanoic acid**	0,180	0,196	0,223	0,188	0,240	0,194	0,230	0,184	0,153	0,000	0,214	0,201	0,198	0,174	0,151	0,234	0,184	0,219	0,162	0,000	0,000	0,000	0,000	0,000	0,000	0,000	0,000	0,000	0,000	0,000	0,000	0,000	0,000	0,000	0,000	0,000	0,000	0,000	0,000	0,000	0,000	0,000	0,000	0,000

Metabolite	Metabolite (IUPAC)	Ser45	Ser46	Ser47	Ser48	Ser49	Ser50	Ser51	Ser52	Ser53	Ser54	Ser55	Ser56	Ser57	Ser58	Ser59	Ser60	Ser61	Ser62	Ser63	Ser64
Citric acid	2-hydroxypropane-1,2,3-tricarboxylic acid	0,000	0,000	0,000	0,000	0,000	0,000	0,000	0,000	0,000	0,000	0,000	0,000	0,000	0,000	0,000	0,000	0,000	0,000	0,000	0,000
L-Lactic acid	(2S)-2-hydroxypropanoic acid	1,490	0,512	0,559	0,571	0,546	0,564	1,328	0,609	1,527	1,636	0,509	0,554	0,455	1,399	1,373	0,545	0,670	1,794	0,611	3,191
L-Alanine	(2S)-2-aminopropanoic acid	0,465	0,000	0,000	0,000	0,000	0,000	1,187	0,000	1,277	0,726	0,000	0,000	0,000	1,519	1,536	0,000	0,000	1,508	0,000	2,480
L-Valine	(2S)-2-amino-3-methylbutanoic acid	0,948	0,955	0,857	1,061	0,922	1,178	1,001	1,192	1,139	1,154	0,942	1,259	1,079	1,268	0,804	0,957	1,629	1,184	1,174	2,158
D-Glucose	(2R,3R,4S,5S,6R)-6-(hydroxymethyl)oxane-2,3,4,5-tetrol	5,298	5,772	4,677	7,052	4,761	5,907	7,913	5,824	8,248	3,437	5,638	6,074	7,321	5,828	8,422	5,073	7,128	8,687	8,008	13,822
L-Leucine	(2S)-2-amino-4-methylpentanoic acid	0,538	0,282	0,815	0,811	0,455	0,359	0,360	0,941	0,641	0,614	0,838	0,354	0,587	0,671	0,826	0,291	0,985	0,390	0,870	1,477
L-Isoleucine	(2S,3S)-2-amino-3-methylpentanoic acid	0,000	0,649	0,273	0,293	0,621	0,614	0,630	0,548	0,343	0,520	0,545	0,000	0,409	0,327	0,283	0,560	1,015	0,000	0,316	1,967
L-Phenylalanine	(2S)-2-amino-3-phenylpropanoic acid	0,186	0,000	0,000	0,000	0,000	0,000	0,288	0,000	0,000	0,297	0,308	0,000	0,000	0,000	0,000	0,205	0,173	0,000	0,000	0,000
Formic acid	formic acid	0,050	0,039	0,035	0,036	0,038	0,043	0,019	0,064	0,032	0,038	0,053	0,036	0,041	0,046	0,050	0,046	0,050	0,049	0,036	0,000
L-Histidine	(2S)-2-amino-3-(1H-imidazol-5-yl)propanoic acid	0,086	0,069	0,064	0,105	0,060	0,092	0,094	0,094	0,063	0,113	0,085	0,117	0,062	0,084	0,055	0,094	0,000	0,072	0,100	0,056
L-Tyrosine	(2S)-2-amino-3-(4-hydroxyphenyl)propanoic acid	0,000	0,240	0,230	0,252	0,236	0,224	0,000	0,230	0,241	0,234	0,252	0,220	0,000	0,232	0,000	0,216	0,099	0,258	0,258	0,000
Choline	(2-hydroxyethyl)trimethylazanium	0,365	0,248	0,349	0,227	0,111	0,167	0,160	0,160	0,182	0,657	0,201	0,172	0,198	0,277	0,000	0,110	0,200	0,227	0,142	0,271
Phosphorylcholine	[2-(trimethylazaniumyl)ethoxy]phosphonic acid	0,070	0,083	0,088	0,078	0,065	0,101	0,101	0,000	0,085	0,063	0,000	0,075	0,068	0,085	0,085	0,071	0,111	0,079	0,080	0,146
Acetone	propan-2-one	0,140	0,110	0,116	0,088	0,081	0,106	0,110	0,123	0,093	0,144	0,090	0,099	0,110	0,093	0,110	0,106	0,150	0,094	0,104	0,170
Acetic acid	acetic acid	0,102	0,080	0,129	0,081	0,061	0,079	0,221	0,063	0,084	0,074	0,067	0,066	0,063	0,094	0,075	0,067	0,075	0,145	0,091	0,883
Acetoacetic acid	3-oxobutanoic acid	0,068	0,062	0,048	0,071	0,064	0,073	0,073	0,082	0,070	0,064	0,070	0,070	0,055	0,075	0,082	0,063	0,094	0,088	0,082	0,000
Methylamine	methanamine	0,069	0,061	0,065	0,047	0,069	0,052	0,076	0,069	0,093	0,045	0,059	0,047	0,047	0,052	0,055	0,062	0,053	0,058	0,059	0,093
Dimethylamine	dimethylamine	0,087	0,063	0,062	0,042	0,059	0,065	0,069	0,129	0,056	0,047	0,000	0,000	0,065	0,061	0,091	0,029	0,072	0,050	0,100	0,000
Creatine	2-(N-methylcarbamimid	0,000	0,065	0,073	0,000	0,066	0,057	0,000	0,000	0,000	0,043	0,044	0,051	0,048	0,060	0,000	0,063	0,076	0,000	0,000	0,000

Metabolite	Metabolite (IUPAC)	Ser65	Ser66	Ser67	Ser68	Ser69	Ser70	Ser71	Ser72	Ser73	Ser74	Ser75	Ser76	Ser77	Ser78	Ser79	Ser80	Ser81	Ser82	Ser83	Ser84	Ser85	Ser86	Ser87	Ser88
Citric acid	2-hydroxypropane-1,2,3-tricarboxylic acid	0,000	0,000	0,000	0,000	0,000	0,000	0,000	0,000	0,000	0,000	0,000	0,000	0,317	0,000	0,000	0,000	0,000	0,000	0,000	0,000	0,000	0,000	0,000	0,000
L-Lactic acid	(2S)-2-hydroxypropanoic acid	0,465	0,592	0,571	1,897	2,073	1,417	2,045	1,299	1,774	1,701	1,684	1,592	0,663	0,727	1,308	0,384	1,583	2,124	1,533	1,448	1,448	2,780	1,576	1,318
L-Alanine	(2S)-2-aminopropanoic acid	0,000	0,000	0,000	1,219	0,650	1,337	0,439	1,384	1,389	1,592	1,613	1,381	0,000	0,000	1,662	0,000	1,290	1,510	1,157	1,317	1,317	1,695	1,688	0,562
L-Valine	(2S)-2-amino-3-methylbutanoic acid	0,865	1,095	1,238	1,098	0,942	1,401	0,828	1,074	1,208	1,201	1,199	1,021	1,163	1,279	1,300	1,063	1,174	1,153	1,016	1,122	1,122	0,805	1,467	0,916
D-Glucose	(2R,3R,4S,5S,6R)-6-(hydroxymethyl)oxane-2,3,4,5-tetrol	3,602	5,702	6,596	7,618	5,478	4,462	5,168	6,390	6,941	7,203	6,740	4,786	5,043	8,832	7,456	6,444	6,551	8,706	7,230	4,735	4,735	11,506	6,036	7,867
L-Leucine	(2S)-2-amino-4-methylpentanoic acid	0,246	0,395	0,376	0,701	0,347	0,757	0,710	0,563	0,657	1,013	0,377	0,348	0,311	0,780	0,843	0,316	0,625	0,531	0,552	0,873	0,873	0,374	0,442	0,300
L-Isoleucine	(2S,3S)-2-amino-3-methylpentanoic acid	0,236	0,765	0,980	0,560	0,303	0,364	0,218	0,000	0,578	0,000	0,000	0,558	0,485	0,473	1,046	0,513	0,000	0,814	0,274	0,298	0,298	0,346	0,383	0,580
L-Phenylalanine	(2S)-2-amino-3-phenylpropanoic acid	0,000	0,000	0,000	0,350	0,313	0,341	0,435	0,318	0,185	0,195	0,188	0,289	0,000	0,086	0,100	0,274	0,327	0,180	0,316	0,314	0,314	0,277	0,266	0,222
Formic acid	formic acid	0,027	0,030	0,041	0,045	0,039	0,033	0,024	0,028	0,030	0,036	0,041	0,054	0,025	0,009	0,003	0,049	0,028	0,037	0,031	0,037	0,037	0,065	0,058	0,049
L-Histidine	(2S)-2-amino-3-(1H-imidazol-5-yl)propanoic acid	0,054	0,101	0,111	0,079	0,058	0,094	0,065	0,116	0,092	0,137	0,110	0,099	0,094	0,036	0,069	0,086	0,096	0,086	0,125	0,092	0,092	0,052	0,103	0,096
L-Tyrosine	(2S)-2-amino-3-(4-hydroxyphenyl)propanoic acid	0,000	0,260	0,000	0,000	0,000	0,223	0,000	0,264	0,229	0,260	0,243	0,217	0,208	0,080	0,203	0,000	0,282	0,229	0,246	0,274	0,274	0,239	0,000	0,232
Choline	(2-hydroxyethyl)trimethylazanium	0,118	0,178	0,144	0,250	0,429	0,103	0,099	0,211	0,256	0,202	0,000	0,000	0,203	0,212	0,205	0,000	0,212	0,163	0,111	0,220	0,220	0,507	0,578	0,218
Phosphorylcholine	[2-(trimethylazaniumyl)ethoxy]phosphonic acid	0,067	0,070	0,000	0,077	0,111	0,000	0,000	0,076	0,071	0,080	0,078	0,068	0,067	0,079	0,038	0,077	0,082	0,089	0,068	0,067	0,067	0,135	0,107	0,000
Acetone	propan-2-one	0,103	0,130	0,147	0,179	0,100	0,142	0,095	0,120	0,097	0,120	0,117	0,108	0,118	0,109	0,147	0,196	0,118	0,122	0,114	0,087	0,087	0,142	0,122	0,092
Acetic acid	acetic acid	0,162	0,066	0,086	0,081	0,137	0,076	0,059	0,082	0,077	0,075	0,100	0,086	0,056	0,222	0,282	0,078	0,071	0,255	0,180	0,138	0,138	0,233	0,076	0,072
Acetoacetic acid	3-oxobutanoic acid	0,059	0,070	0,076	0,076	0,087	0,068	0,103	0,075	0,081	0,089	0,072	0,204	0,073	0,056	0,043	0,061	0,077	0,089	0,089	0,084	0,084	0,207	0,082	0,063
Methylamine	methanamine	0,068	0,075	0,071	0,044	0,050	0,045	0,055	0,046	0,041	0,056	0,053	0,065	0,040	0,092	0,043	0,065	0,048	0,069	0,062	0,048	0,048	0,074	0,053	0,040
Dimethylamine	dimethylamine	0,118	0,064	0,085	0,049	0,075	0,051	0,062	0,038	0,052	0,053	0,035	0,039	0,052	0,122	0,270	0,064	0,057	0,131	0,053	0,071	0,071	0,197	0,081	0,000
Creatine	2-(N-methylcarbamimid	0,00	0,00	0,000	0,000	0,05	0,00	0,00	0,00	0,00	0,05	0,00	0,05	0,00	0,00	0,00	0,00	0,07	0,00	0,00	0,07	0,07	0,000	0,00	0,00

	**amido)acetic acid**																					0	0			3	0	0	0	0	7	0	0	0	0	0	0	4	0	0	2	2		0	0
**Creatinine**	**2-imino-1-methylimidazolidin-4-one**	0,142	0,141	0,151	0,156	0,160	0,219	0,000	0,170	0,145	0,222	0,165	0,161	0,151	0,144	0,117	0,185	0,115	0,246	0,134	0,219	0,200	0,182	0,167	0,152	0,169	0,225	0,117	0,163	0,132	0,231	0,162	0,185	0,142	0,000	0,000	0,173	0,082	0,000	0,138	0,148	0,148	0,284	0,237	0,143
**Glycine**	**2-aminoacetic acid**	0,355	0,232	0,228	0,198	0,245	0,215	0,594	0,172	0,204	0,290	0,191	0,237	0,257	0,277	0,236	0,210	0,266	0,173	0,149	0,000	0,199	0,236	0,229	0,309	0,306	0,318	0,197	0,292	0,281	0,283	0,267	0,272	0,233	0,000	0,000	0,279	0,320	0,000	0,320	0,281	0,281	0,313	0,341	0,246
**Methanol**	**methanol**	0,000	0,000	0,161	0,255	0,000	0,000	0,000	0,184	0,000	0,128	0,233	0,000	0,000	0,000	0,000	0,133	0,207	0,000	0,000	0,000	0,000	0,000	0,000	0,000	0,000	0,000	0,000	0,147	0,000	0,147	0,000	0,000	0,000	0,000	0,000	0,000	0,000	0,000	0,119	0,000	0,000	0,000	0,000	0,000
**Betaine**	**(carboxymethyl)trimethylazanium**	0,059	0,090	0,112	0,073	0,060	0,083	0,084	0,063	0,083	0,075	0,053	0,080	0,063	0,077	0,086	0,057	0,081	0,074	0,067	0,276	0,077	0,073	0,054	0,082	0,053	0,047	0,036	0,049	0,094	0,057	0,082	0,054	0,065	0,101	0,069	0,071	0,094	0,090	0,062	0,073	0,073	0,112	0,078	0,045
**3-Hydroxybutyric acid**	**(3R)-3-hydroxybutanoic acid**	0,146	1,178	1,046	1,100	1,203	1,349	0,303	1,691	0,000	0,280	1,367	1,344	1,021	0,776	0,000	1,258	1,456	0,000	1,500	0,000	1,357	1,490	1,464	0,236	0,122	0,115	0,113	0,000	0,103	0,306	0,309	0,000	1,450	1,703	0,000	1,134	0,621	0,000	0,108	0,506	0,506	0,168	0,000	0,117
**2-Hydroxybutyric acid**	**(2S)-2-hydroxybutanoic acid**	0,000	0,000	0,000	0,000	0,000	0,000	0,000	0,000	0,000	0,000	0,000	0,000	0,000	0,000	0,000	0,000	0,000	0,000	0,000	0,000	0,000	0,000	0,000	0,000	0,000	0,000	0,000	0,000	0,000	0,000	0,000	0,000	0,000	0,000	0,000	0,000	0,000	0,000	0,000	0,000	0,000	0,000	0,000	0,000
**Isobutyric acid**	**2-methylpropanoic acid**	0,862	0,413	0,472	0,365	0,399	0,000	0,000	0,492	0,339	0,291	0,000	0,283	0,272	0,308	0,187	0,361	0,235	0,000	0,333	0,495	0,000	0,464	0,419	0,271	0,596	0,518	0,444	0,842	0,581	0,699	0,624	0,542	0,000	0,000	0,241	0,750	0,454	0,000	0,333	0,729	0,729	0,754	0,272	0,799
**(S)-3-Hydroxyisobutyric acid**	**(2S)-3-hydroxy-2-methylpropanoic acid**	0,375	0,000	0,000	0,000	0,431	0,000	0,000	0,000	0,000	0,000	0,000	0,000	0,000	0,453	0,000	0,000	0,000	0,000	0,000	0,000	0,000	0,000	0,000	0,273	0,000	0,000	0,000	0,000	0,000	0,000	0,000	0,000	0,000	0,000	0,000	0,359	0,000	0,000	0,000	0,000	0,000	0,000	0,000	0,000
**L-Glutamine**	**(2S)-2-amino-4-carbamoylbutanoic acid**	0,615	1,140	0,901	0,578	0,609	0,632	0,639	0,907	0,335	0,584	1,186	1,298	0,879	1,029	1,050	1,240	0,835	1,113	0,896	0,000	0,725	0,967	1,124	0,325	0,318	0,964	0,275	0,987	0,715	1,145	1,045	0,955	0,605	0,000	0,000	0,309	0,663	0,186	0,960	0,650	0,650	0,721	0,386	0,519
**L-Glutamic acid**	**(2S)-2-aminopentanedioic acid**	0,654	0,000	0,000	0,565	0,576	0,000	0,000	0,000	0,000	0,666	0,000	0,510	0,000	0,000	0,000	0,000	0,743	0,000	0,000	1,110	0,000	0,000	0,000	0,000	0,000	0,000	0,000	0,000	0,775	0,000	0,000	0,000	0,000	0,000	1,164	0,000	0,564	0,000	0,000	0,761	0,761	0,000	0,000	0,000
**L-Aspartic acid**	**(2S)-2-aminobutanedioic acid**	0,000	0,000	0,000	0,000	0,000	0,000	0,000	0,000	0,000	0,000	0,000	0,000	0,000	0,000	0,000	0,000	0,000	0,000	0,000	0,000	0,000	0,000	0,000	0,000	0,000	0,000	0,000	0,000	0,437	0,000	0,000	0,000	0,000	0,000	0,000	0,000	0,406	0,000	0,000	0,000	0,000	0,000	0,000	0,000
**L-Asparagine**	**(2S)-2-amino-3-carbamoylpropanoic acid**	0,306	0,000	0,000	0,000	0,000	0,000	0,000	0,000	0,000	0,000	0,000	0,000	0,000	0,000	0,225	0,144	0,000	0,000	0,000	0,000	0,000	0,320	0,000	0,000	0,000	0,191	0,000	0,426	0,214	0,306	0,000	0,170	0,275	0,000	0,388	0,000	0,268	0,000	0,000	0,000	0,000	0,000	0,298	0,000
**L-Threonine**	**(2S,3R)-2-amino-3-hydroxybutanoic acid**	0,207	0,242	0,392	0,508	0,295	0,515	0,491	0,525	0,000	0,458	0,284	0,493	0,479	0,271	0,344	0,361	0,255	0,246	0,361	0,666	0,302	0,411	0,537	0,248	0,220	0,264	0,265	0,440	0,452	0,462	0,399	0,369	0,364	0,294	0,511	0,419	0,406	0,387	0,267	0,512	0,512	0,166	0,381	0,436
**D-Lysine**	**(2R)-2,6-diaminohexanoic acid**	0,000	0,000	0,000	0,000	0,000	0,000	0,522	0,000	0,277	0,261	0,000	0,000	0,000	0,301	0,000	0,000	0,000	0,000	0,000	1,257	0,000	0,000	0,000	0,292	0,000	0,294	0,000	0,000	0,275	0,000	0,000	0,346	0,000	0,000	0,857	0,000	0,273	0,849	0,000	0,000	0,000	0,351	0,359	0,000
**L-Arginine**	**(2S)-2-amino-5-carbamimidamidopentanoic acid**	0,000	0,000	0,000	0,000	0,000	0,000	0,895	0,000	0,765	1,159	0,000	0,000	0,000	0,344	0,000	0,000	0,000	0,000	0,000	2,756	0,000	0,000	0,000	0,000	0,000	0,935	0,000	0,000	1,526	0,000	0,000	1,090	0,000	0,000	2,107	0,000	0,944	1,298	0,000	0,000	0,000	2,140	1,387	0,000
**L-Methionine**	**(2S)-2-amino-4-(methylsulfanyl)butanoic acid**	0,000	0,000	0,000	0,000	0,000	0,000	0,000	1,207	0,000	0,000	0,000	0,000	0,000	0,000	0,000	0,000	0,000	0,000	0,000	0,000	0,000	0,000	0,000	0,000	0,000	0,000	0,000	0,000	0,000	0,000	0,000	0,000	0,000	0,000	0,000	0,000	0,000	0,000	0,000	0,000	0,000	0,000	0,000	0,000
**Syringic acid**	**4-hydroxy-3,5-dimethoxybenzoic acid**	0,000	0,000	0,000	0,000	0,000	0,000	0,000	0,000	0,000	0,000	0,000	0,000	0,000	0,000	0,000	0,000	0,000	0,000	0,000	0,000	0,000	0,000	0,000	0,000	0,000	0,000	0,000	0,000	0,000	0,000	0,000	0,000	0,000	0,000	0,000	0,000	0,000	0,000	0,000	0,000	0,000	0,000	0,000	0,000
**Pyruvic acid**	**2-oxopropanoic acid**	0,000	0,000	0,000	0,000	0,000	0,000	0,000	0,000	0,000	0,000	0,000	0,000	0,000	0,000	0,137	0,000	0,000	0,000	0,000	0,000	0,000	0,000	0,000	0,000	0,000	0,000	0,000	0,000	0,000	0,000	0,000	0,000	0,000	0,000	0,000	0,000	0,000	0,000	0,000	0,000	0,000	0,000	0,000	0,000

Table S6 A summary of the identified metabolites

Metabolite	Metabolite (IUPAC)	Chemical shifts	Assignment	Multiplicity
Valine	(2S)-2-amino-3-methylbutanoic acid	0.99, 1.03	CH_3, CH_3	d, d
Lactate	(2S)-2-hydroxypropanoic acid	1.33, 4.11	CH_3, CH	d, q
Glucose	(2R,3R,4S,5S,6R)-6-(hydroxymethyl)oxane-2,3,4,5-tetrol	3.4-4.0, 5.23-	various, H1, CH	m, d
Leucine	(2S)-2-amino-4-methylpentanoic acid	0.96	d-CH_3	d+d
Isoleucine	(2S,3S)-2-amino-3-methylpentanoic acid	0.93, 1.00	d-CH_3, b-CH_3	t, d
Alanine	(2S)-2-aminopropanoic acid	1.47	CH_3	d
Creatine	2-(N-methylcarbamimidamido)acetic acid	3.04, 3.93	CH_3, CH_2	s, s
1-Methyl histidine,	1-methyl-L-histidine	7.06, 7.79	CH_2, CH_2	s, s
Tyrosine	(2S)-2-amino-3-(4-hydroxyphenyl)propanoic acid	6.88, 7.17	CH, CH	dd, dd
Formic acid	Formic acid	8.45	CH	s
Phenyl alanine	2S)-2-amino-3-phenylpropanoic acid	7.33, 7.43	H2 + H6, H3+ H5	m, m
Glutamine	(2S)-2-amino-4-carbamoylbutanoic acid	2.44	half g-CH_2	m
Acetoacetate	3-oxobutanoic acid	2.23	CH_3	s
Glutamic acid	(2S)-2-aminopentanedioic acid	2.35	half g-CH_2	m
Acetic acid	Acetic acid	1.92	CH_3	s
Acetone	propan-2-one	2.24	CH_4	s
Citric acid	2-hydroxypropane-1,2,3-tricarboxylic acid	2.53, 2.69	half CH_2, half CH_2	d, d
Choline	(2-hydroxyethyl)trimethylazanium	3.21	$N(CH_3)_3$	s
Creatinine	2-imino-1-methylimidazolidin-4-one	3.05, 4.05	CH_3, CH_2	s, s
Dimethylamine	Dimethylamine	2.9		s
L-Aspartic acid	(2S)-2-aminobutanedioic acid	2.68, 2.82, 3.90	half CH2, half CH2, CH	dd, dd,dd
L-Asparagine	(2S)-2-amino-3-carbamoylpropanoic acid	2.85, 2.95, 4.00	half β-CH2, half β-CH2, α-CH	m, m, dd
3-Hydroxybutyric acid	(3R)-3-hydroxybutanoic acid	2.30, 2.40	half a-CH_2, half a-CH_2	m, m
Glycine	2-aminoacetic acid	3.55	CH_2	s
Betaine	(carboxymethyl)trimethylazanium	3,28	CH_2	s

L-Threonine	(2S,3R)-2-amino-3-hydroxybutanoic acid	1.33, 3.59, 4.25	γ-CH3, α-CH, β-CH	d, d, m
D-Lysine	(2R)-2,6-diaminohexanoic acid	1.68-1.75	d-CH_2, g-CH_2	m, m
cholesterol-VLDL	(3β)-cholest-5-en-3-ol-VLDL	0,72	R–CH 3	m
LDL2/VLDL2	LDL2/VLDL2	1.22-1.30	$(CH_2)_n/CH_2CH_2CH_2CO$	m
unsaturated lipid	unsaturated lipid	5,15	–CH]CH–	m
glycerophosphocholine	(2-{[(2R)-2,3-dihydroxypropyl phosphono]oxy}ethyl)trimethylazanium	3.22, 3.68, 4.32	CH3, NCH2, OCH2CH2	s, t, t
O-Phosphocholine	[2-(trimethylazaniumyl)ethoxy]phosphonic acid	3,24		s
